# Supplementary material for: Dynamic FMR1 granule phase switch instructed by m6A modification contributes to maternal RNA decay
Source: Nat Commun. 2022 Feb 14;13:859. doi: 10.1038/s41467-022-28547-7 (PMC8844045; doi:10.1038/s41467-022-28547-7)
Supplement: Supplementary file 1 — Supplementary Information [file 41467_2022_28547_MOESM1_ESM.pdf]

Supplementary Information

Dynamic FMR1 granule phase switch instructed by m6A modification contributes to maternal RNA decay

Guoqiang Zhang[1, 4], Yongru Xu[1, 2, 4], Xiaona Wang[2, 4], Yuanxiang Zhu[1, 2, 4], Liangliang Wang[1], Wenxin Zhang[1], Yiru Wang[1], Yajie Gao[1,2], Xuna Wu[3], Ying Cheng[1], Qinmiao Sun[2]* and Dahua Chen[1, 2]*

[1] Institute of Biomedical Research, Yunnan University, Kunming, China

[2] State Key Laboratory of Membrane Biology, Institute of Zoology, Chinese Academy of Sciences, Beijing, China

[3] School of Life Sciences, Yunnan University, Kunming, China

[4] These authors contributed equally to this work.

Supplementary Figures

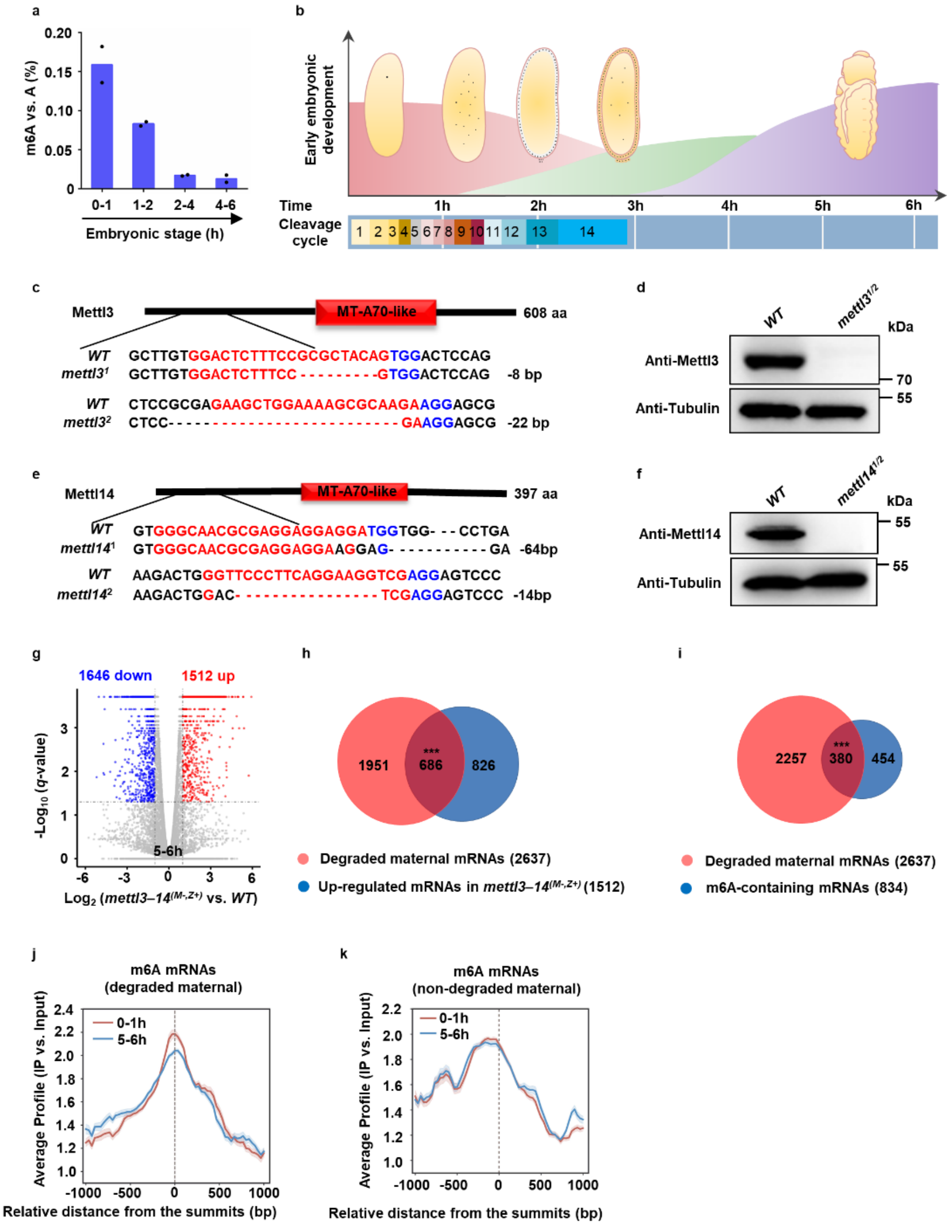

Figure S1. **The m6A modification regulates maternal RNA decay. a**, Abundances of m6A on mRNAs from indicated embryos were measured by mass spectrometry. The mole ratios of m6A vs. A are shown on the y axis. Data expressed as means of two independent samples. **b**, A comparative overview of early embryonic development in *Drosophila*. Timeline of the early embryogenesis are depicted schematically above. The red curves represent the degradation profiles of destabilized maternal transcripts. The green and purple curves illustrate the zygotic

genome activation. **c**, Schematic representation of *mettl3* mutant allele generation using the CRISPR/Cas9 system. **d**, Western blot assays showed that the Mettl3 protein was completely abolished in *mettl3* mutant flies. Representative figures of two independent replicates are shown. **e**, Schematic representation of *mettl14* mutant allele generation using the CRISPR/Cas9 system. **f**, Western blot assays showed that the Mettl14 protein was completely abolished in *mettl14* mutant flies. Representative figures of two independent replicates are shown. **g**, Volcano plot showing transcriptome-wide $\log_2$ fold changes in wide-type and *mettl3-mettl14* maternal mutant embryos. Upregulated and downregulated genes are highlighted in red and blue, respectively. **h**, Overlap of degraded maternal group and up-regulated transcripts in *mettl3-mettl14* maternal mutant embryos. P-value (1.08e-69) was calculated by one-sided hypergeometric test. $^{***}P < 0.001$. **i**, Overlap of degraded maternal mRNAs and m6A marked mRNAs in 0–1-hour embryos. P-value (4.3e-09) was calculated by one-sided hypergeometric test. $^{***}P < 0.001$. **j** and **k**, The average m6A signal profiles for degraded (**j**, P = 1.75e-22) and non-degraded (**k**, P = 0.084) maternal mRNAs marked with m6A at the 0–1-hour and 5–6-hour stage embryos. The statistically significant was estimated by comparing the values representing the read counts of m6A after normalization in the ten bins around the summit (~ ± 300 bp) using two-sided wilcoxon rank-sum test. Error bars indicate mean ± 1.96 SE. Source data are provided as a Source Data file and Supplementary Data 1-3.

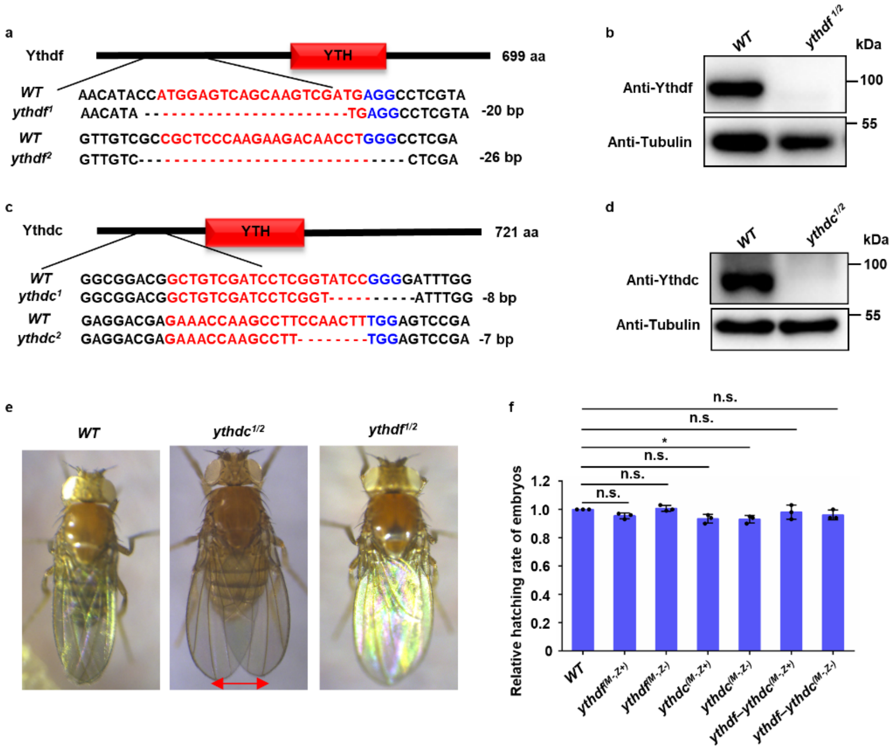

Figure S2. **The m6A regulates maternal RNA decay independently of YTH factors. a**, Schematic representation of *ythdf* mutant allele generation using the CRISPR/Cas9 system. **b**, Western blot assays showed that the Ythdf protein was completely abolished in *ythdf* mutant flies. Representative figures of two independent replicates are shown. **c**, Schematic representation of *ythdc* mutant allele generation using the CRISPR/Cas9 system. **d**, Western blot assays showed that the Ythdc protein was completely abolished in *ythdc* mutant flies. Representative figures of two independent replicates are shown. **e**, Wild-type and *ythdf* mutant flies have their wings properly folded, while *ythdc* mutant flies cannot fold their wings and exhibit a mildly held-out wing phenotype (marked by the double arrows). **f**, Relative hatching rate of embryos with indicated genotypes ($ythdf^{(M-,Z+)}$, P = 0.06; $ythdf^{(M-,Z-)}$, P = 0.63; $ythdc^{(M-,Z+)}$, P = 0.063; $ythdc^{(M-,Z-)}$, P = 0.044; $ythdf\text{-}ythdc^{(M-,Z+)}$, P = 0.56; $ythdf\text{-}ythdc^{(M-,Z-)}$, P = 0.18). The data expressed as means of three independent experiments, and the two-sided Student's t-test was used to analyze statistical variance. Error bars indicate mean ± SD. *P < 0.05, n.s., not significant. Source data are provided as a Source Data file.

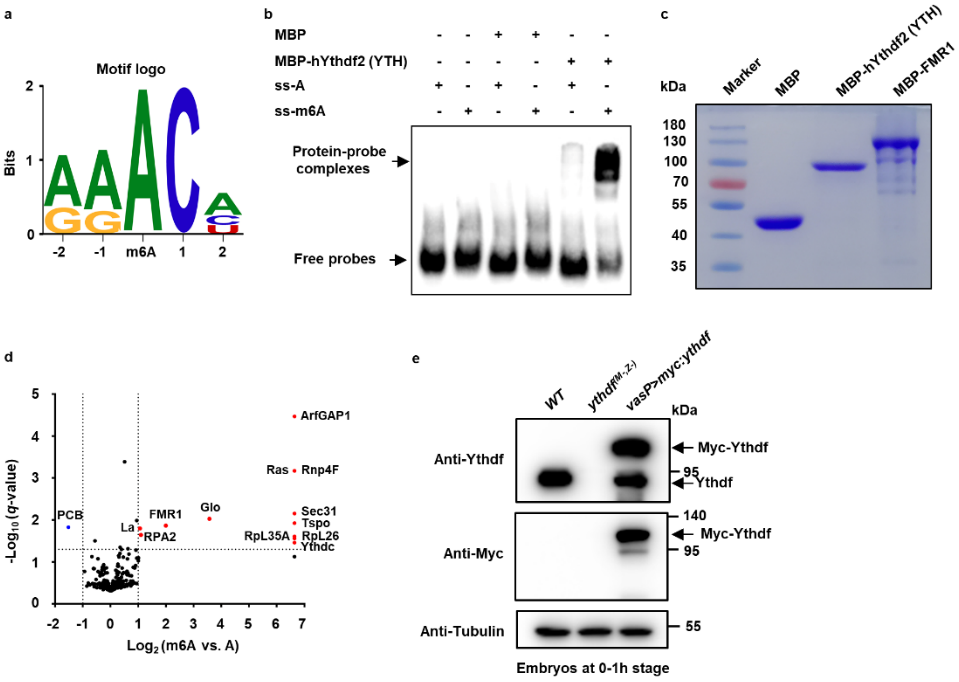

Figure S3. **Identifying FMR1 as an m6A modified RNA-binding protein in *Drosophila* embryos. a**, Logo representations of consensus motifs within HC-m6A peaks at the 0–1-hour embryonic stage in wild-type embryos (P = 0.012). **b**, EMSAs showing the binding affinities of human Ythdf2 (hYthdf2, 300-579 aa) with m6A modified and unmodified probes. Representative figures of two independent replicates are shown. **c**, Coomassie brilliant blue staining of purified proteins as indicated. The experiment was performed once. **d**, Volcano plot showing interactome changes in complexes immuno-precipitated by the unmodified and m6A-modified probes. The x-axis shows $\log_2$ fold changes of m6A-modified probes compared with unmodified probes. The y-axis shows $\log_2$ q-value of fold changes of m6A-modified probes compared with unmodified probes. The experiments were performed in three biological replicates. The one-sided Student's t-test was used to analyze statistical significance. For preferentially interacting proteins, they were defined as those with at least 2-fold upregulated (q-value < 0.05) in m6A-modified probe samples. **e**, The Ythdf antibody was validated by western blot assays. Representative figures of two independent replicates are shown. Source data are provided as a Source Data file and Supplementary Data 3, 4.

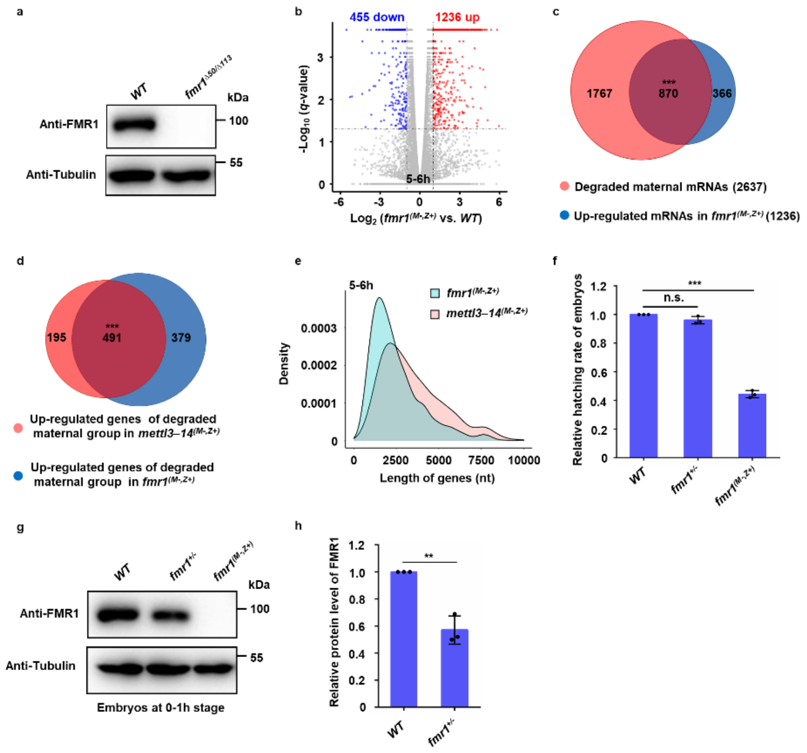

Figure S4. **FMR1 regulates maternal RNA decay at least in part via m6A modification. a**, FMR1 protein was absent in *fmr1* mutant flies. Representative figures of two independent replicates are shown. **b**, Volcano plot showing transcriptome-wide $\log_2$ fold changes in wide-type and *fmr1* maternal mutant embryos. Upregulated and downregulated genes are highlighted in red and blue, respectively. **c**, Overlap of degraded maternal group and up-regulated transcripts in *fmr1* maternal mutant embryos. P-value (1.7e-271) was calculated by one-sided hypergeometric test. **d**, Overlap of aberrantly up-regulated transcripts in degraded maternal group between *fmr1* maternal mutant and *mettl3-mettl14* double maternal mutant embryos. P-value (9.8e-54) was calculated by one-sided hypergeometric test. **e**, Distributions of read density over degraded maternal gene length that up-regulated in *fmr1* and *mettl3-mettl14* double maternal mutants at the 5-6 hour time point. **f**, Relative hatching rate of embryos with indicated genotypes ($fmr1^{+/-}$, P = 0.059; $fmr1^{(M-,Z+)}$, P = 2.8e-06). **g**, Western blot assays were performed to show expression levels of endogenous FMR1 in the 0–1-hour stage embryos with indicated genotypes. **h**, The quantitative intensity of bands boxed in the panel **g** (P = 0.002). In **f** and **h**, the two-sided Student's t-test was used to analyze statistical variance. Data expressed as means of three independent experiments. Error bars indicate mean ± SD. **P < 0.01, ***P < 0.001. n.s., not significant. Source data are provided as a Source Data file and Supplementary Data 1, 2.

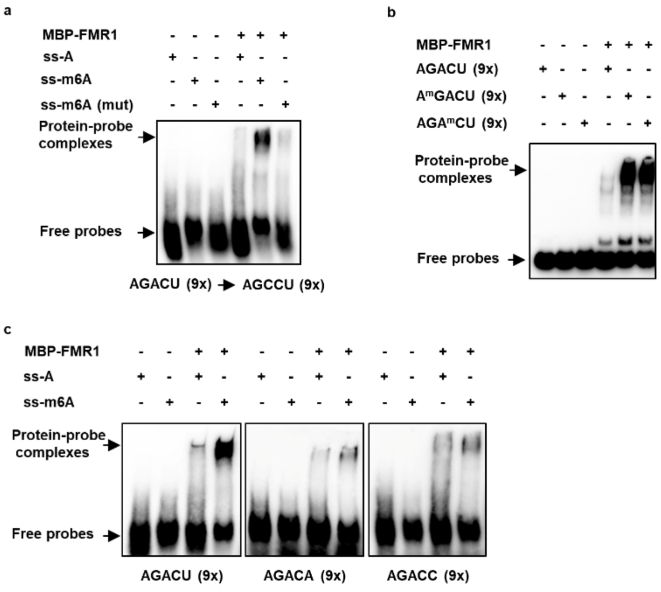

Figure S5. **FMR1 preferentially binds the m6A-marked RNA containing the "AGACU" motif. a**, EMSAs showing the binding capability of FMR1 to RNA probes containing repeated "AGACU" motif or "AGCCU" mutant motif with or without m6A modification. **b**, EMSAs showing the binding capability of FMR1 to the "A^{m}GACU" or "AGA^{m}CU"-containing probes, in which only the first A or the central A was modified by m6A. **c**, EMSAs showing the binding capability of FMR1 to RNA probes containing either "AGACU", "AGACA" or "AGACC" sequences (9x) with or without m6A modification. In **a**-**c**, representative figures of three independent replicates are shown. Source data are provided as a Source Data file.

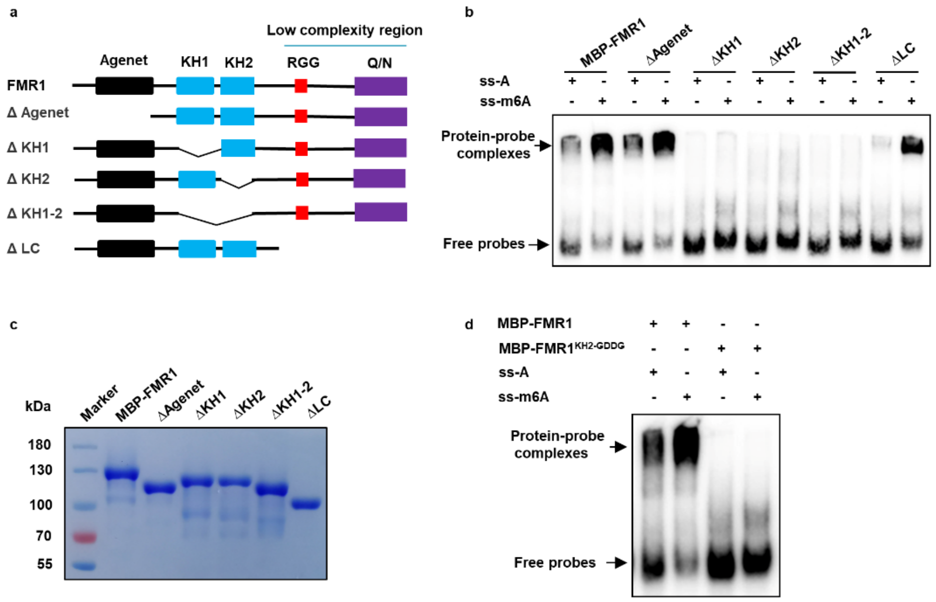

Figure S6. **The "GxxG" loop within KH domains is required for FMR1 to bind the RNA probes. a**, Schematic drawings of FMR1 and its deletion mutants. **b**, EMSAs showing the binding capability of truncated FMR1 proteins to m6A modified and unmodified RNA probes. Representative figures of three independent replicates are shown. **c**, Coomassie brilliant blue staining of purified proteins. The experiment was performed once. **d**, EMSAs showing the binding capability of FMR1$^{KH2\text{-}GDDG}$ protein to m6A modified and unmodified RNA probes. Representative figures of three independent replicates are shown. Source data are provided as a Source Data file.

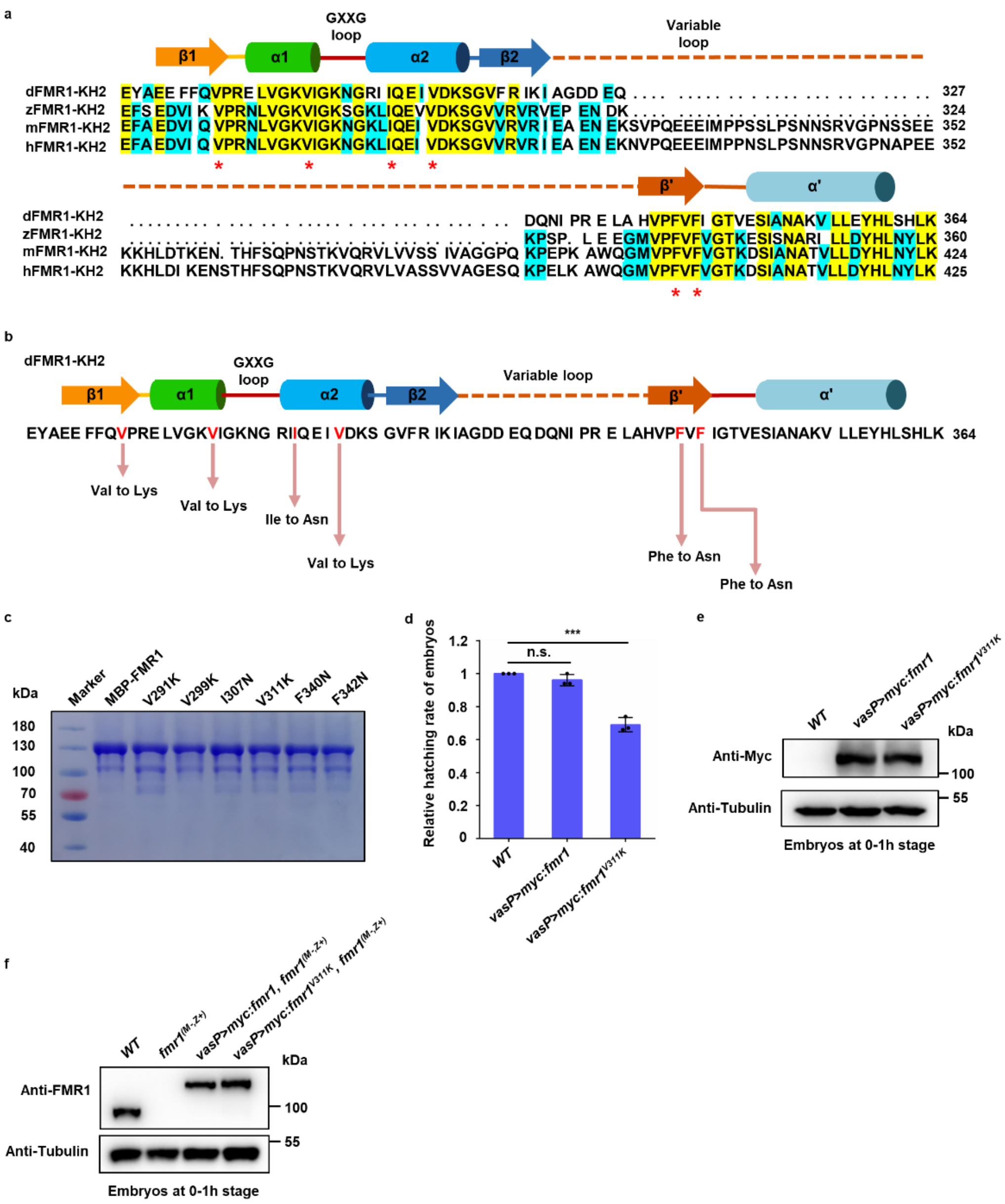

Figure S7. FMR1 regulates decay of its target maternal mRNAs in m6A-dependent manner. a, Alignment of FMR1 amino acid sequence with its homolog for conserved KH2 domain. **b**, Schematic diagram displaying amino acid mutation site and amino acid being mutated in KH2 domain of FMR1. **c**, Coomassie brilliant blue staining of purified proteins. **d**, Relative hatching rate of embryos with indicated genotypes at 29°C (*vasP>myc:fmr1*, P = 0.12; *vasP>myc:fmr1*^{V311K}, P = 0.0003). The two-sided Student's t-test was used to analyze statistical variance. Data expressed as means of three independent experiments. Error bars indicate mean ± SD. ***P < 0.001. n.s., not significant. **e**, Western blot assays were performed to show expression levels of overexpressed Myc-FMR1 or Myc-FMR1^{V311K} in the 0–1-hour embryos

with indicated genotypes. **f**, Western blot assays were performed to show expression levels of endogenous FMR1, overexpressed Myc-FMR1 and Myc-FMR1^{V311K} in 0–1-hour stage embryos with indicated genotypes. In **c**, **e**, and **f**, the experiment was performed once. Source data are provided as a Source Data file.

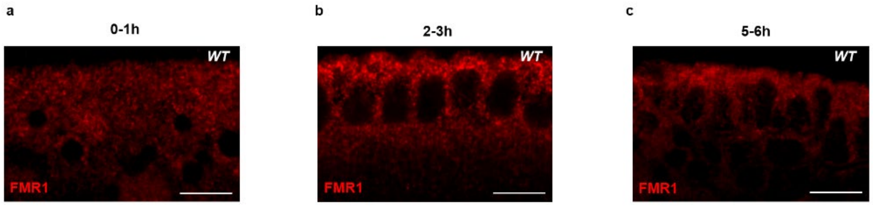

Figure S8. **FMR1 granules undergo a dynamical assembly in early embryos.**

a-c, Wild-type embryos at the 0–1-hour (**a**), 2–3-hour (**b**) and 5–6-hour (**c**) stages were stained with anti-FMR1 (red) antibody. Representative figures of three independent replicates are shown. Scale bars, 10 μm.

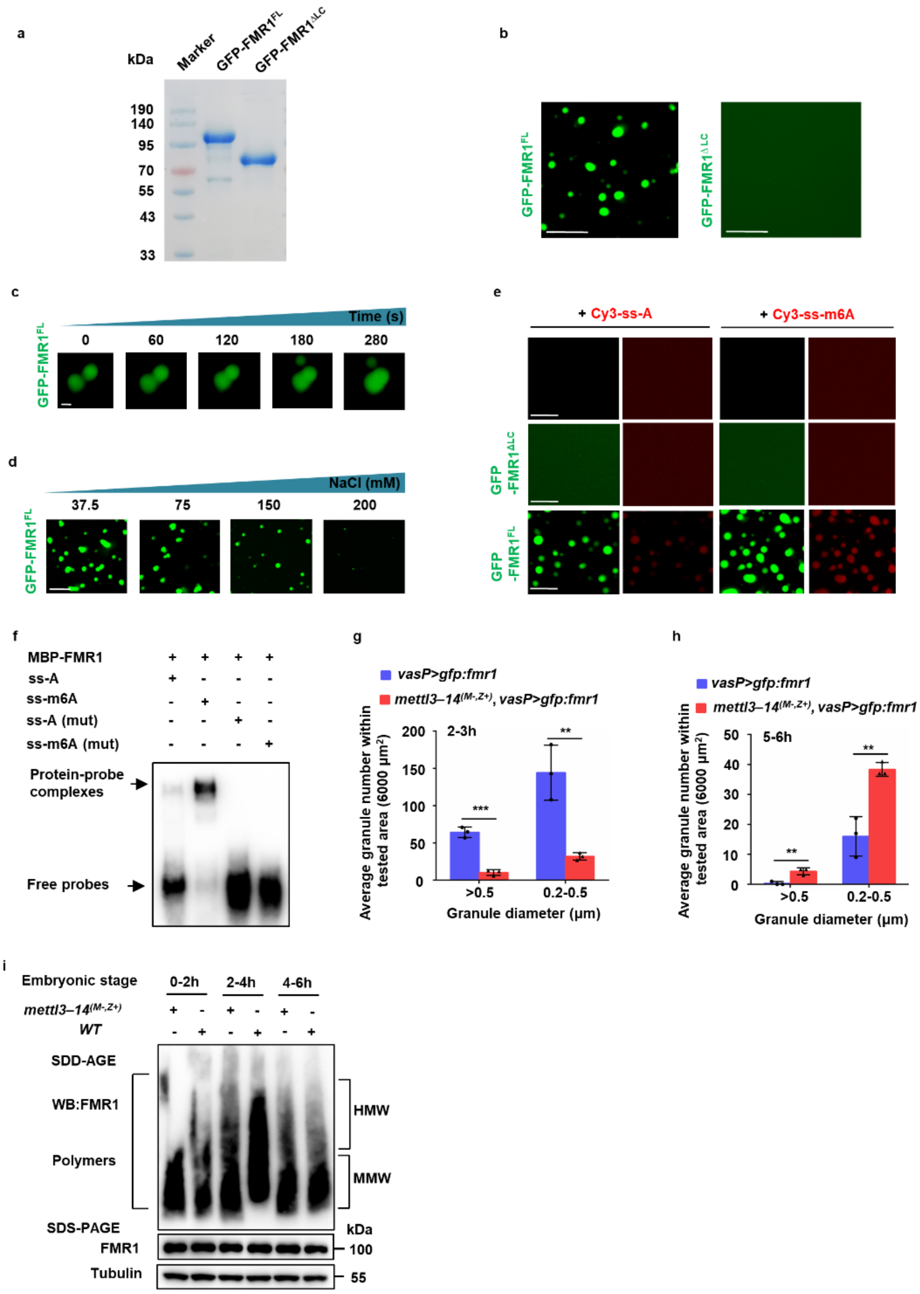

Figure S9. **The m6A modification regulates assembly/disassembly switch of FMR1 granules.** **a**, Coomassie brilliant blue staining of purified proteins. The experiment was performed once. **b**, *in vitro* phase separation assays were performed to detect GFP-FMR1^{FL} or GFP-FMR1$^{\Delta LC}$ (10 uM) droplet formation. Scale bars, 10 μm. **c**, The small sized droplets of GFP-FMR1^{FL} (10 uM) underwent fusion to form larger ones. Scale bars, 1 μm. **d**, Representative images of phase separation for GFP-FMR1^{FL} (10 uM) with increased dose of salt (NaCl). Scale bars, 10 μm. **e**, Droplet formation for GFP-FMR1$^{\Delta LC}$ (10 uM) mixed with

m6A modified and unmodified RNA probes. Scale bars, 10 µm. **f**, EMSAs showing the binding capability of FMR1 to RNA probes (repeated "GGACU", "AGACU" and "GAACU" motif) or mutant RNA probes (repeated "GGCCU", "AGCCU" and "GACCU" motif) with or without m6A modification. **g** and **h**, Quantifying the average number of FMR1 granules with different sizes within tested area (Fig. 4i) (**g**: >0.5, P = 0.0003; 0.2-0.5, P = 0.0065. **h**: >0.5, P = 0.0058; 0.2-0.5, P = 0.0051). The two-sided Student's t-test was used to analyze statistical variance. Data expressed as means of 3 independent experiments. Error bars indicate mean ± SD. **P < 0.01, ***P < 0.001. **i**, Crude embryo extracts of wild-type and *mettl3-mettl14* double maternal mutants at indicated embryonic stages were prepared, and then aliquots of the extracts were analyzed by SDD-AGE and SDS-PAGE assays. Representative figures of two independent replicates are shown. HMW, high-molecular weight, MMW, middle-molecular weight. In **b**-**f**, representative figures of three independent replicates are shown. Source data are provided as a Source Data file.

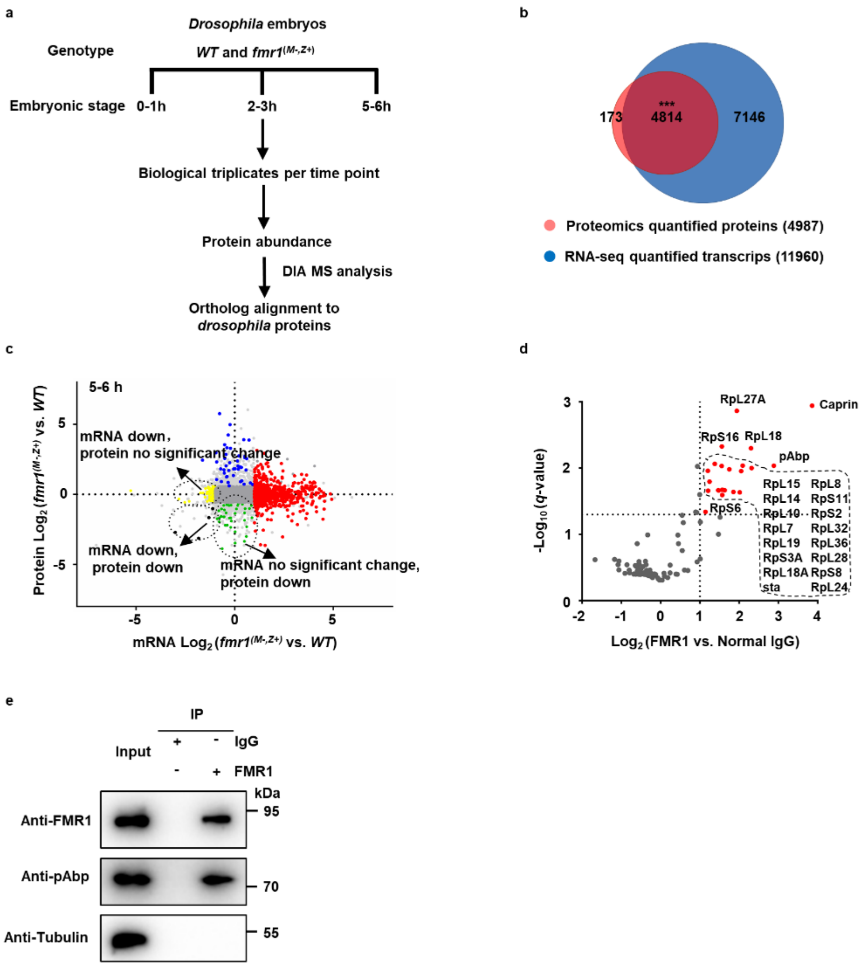

Figure S10. **FMR1 granule phase-switch regulates maternal RNA decay in early embryos.** **a**, Schematic for a data independent acquisition (DIA) proteomics approach on the wild-type and *fmr1* maternal mutant embryos at multiple developmental stages. **b**, Overlap of proteomics quantified proteins and RNA-seq quantified transcripts in *fmr1* maternal mutant embryos. P-value (0) was calculated by one-sided hypergeometric test. ***P < 0.001. **c**, Scatterplot integrating protein and mRNA levels (4814 genes)were generated by using wild-type and *fmr1* maternal mutant embryos at the 5–6-hour stage. In addition to Group1-3 (Fig. 5a), other groups were classified: the genes with downregulated mRNA level, but limited protein level change (yellow); the genes with downregulated mRNA and protein level (black); the genes with downregulated protein level, but limited mRNA level change (green). **d**, Volcano plot showing interactome changes in complexes immuno-precipitated by FMR1 antibody. The x-axis shows $\log_2$ fold changes of FMR1 antibody compared with control IgG. The y-axis shows $\log_2$ q-value of fold changes of FMR1 antibody compared with control IgG. The experiments were

performed in three biological replicates. The one-sided Student's t-test was used to analyze statistical significance. Proteins that were upregulated more than 2-fold (q-value < 0.05) in three biological replicates compared with control IgG were considered as FMR1-associated proteins. **e**, Coimmunoprecipitation of FMR1 with pAbp in wide-type embryos at 2–4-hour embryonic stage. The lysates were immunoprecipitated with anti-FMR1 antibody, and western blot assays were performed to detect pAbp protein in each immunoprecipitation. Tubulin was used as the negative control. Representative figures of three independent replicates are shown. Source data are provided as a Source Data file and Supplementary Data 4.

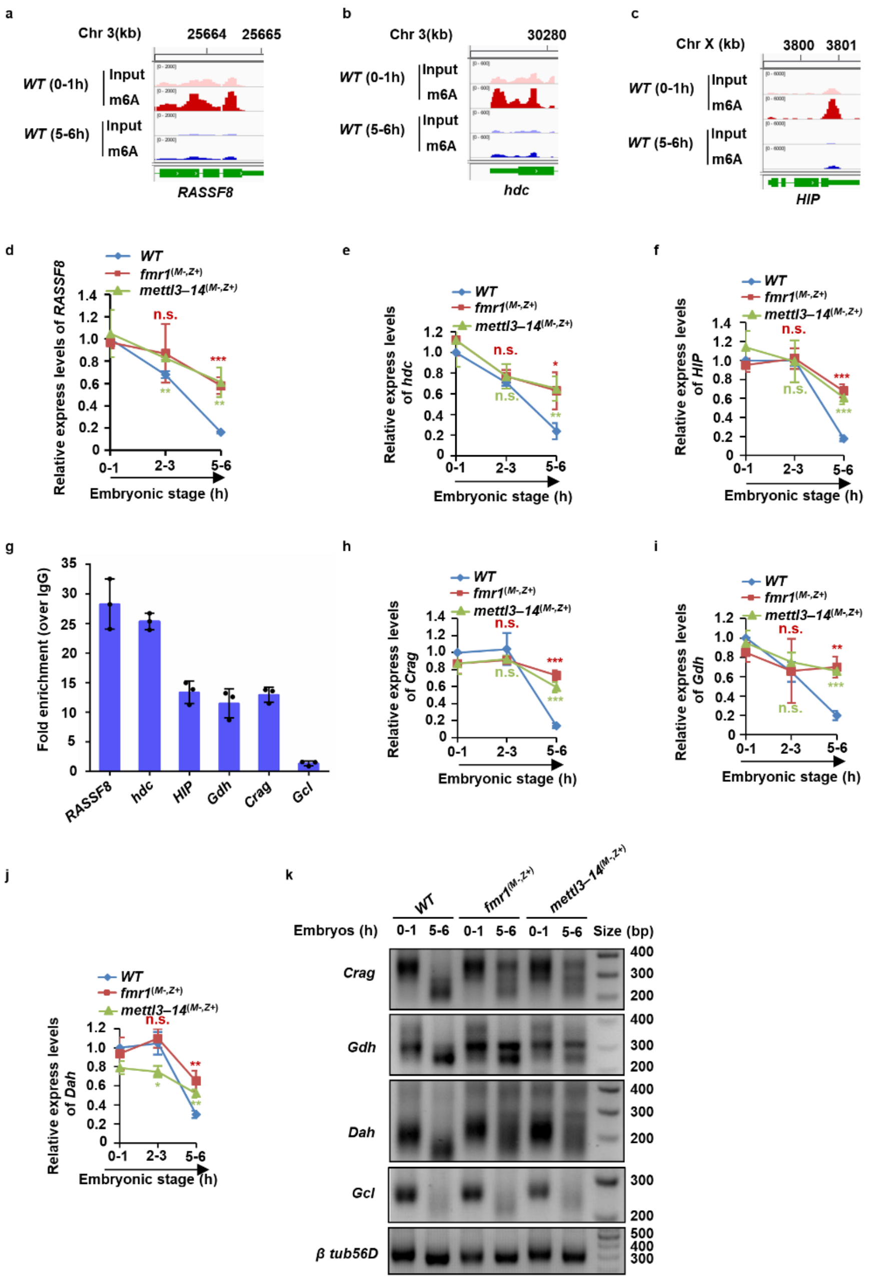

Figure S11. FMR1 regulates decay of its target maternal mRNAs in m6A-dependent manner. a-c, IGV tracks displaying MeRIP-m6A-seq read distribution in *RASSF8* (**a**)*, hdc* (**b**) and *HIP* (**c**) mRNAs in wild-type embryos at the 0–1-hour and 5–6-hour stages. **d-f,** q-RT-PCR assays showing expression levels of *RASSF8* (**d**) (2-3h: *fmr1*$^{(M-,Z+)}$, P = 0.3; *mettl3-14*$^{(M-,Z+)}$, P = 0.0019. 5-6h: *fmr1*$^{(M-,Z+)}$, P = 0.0007; *mettl3-14*$^{(M-,Z+)}$, P = 0.0046), *hdc* (**e**) (2-3h: *fmr1*$^{(M-,Z+)}$, P = 0.19; *mettl3-14*$^{(M-,Z+)}$, P = 0.45. 5-6h: *fmr1*$^{(M-,Z+)}$, P = 0.028; *mettl3-14*$^{(M-,Z+)}$, P = 0.0091) and *HIP* (**f**) (2-3h: *fmr1*$^{(M-,Z+)}$, P = 0.83; *mettl3-14*$^{(M-,Z+)}$, P = 0.92. 5-6h: *fmr1*$^{(M-,Z+)}$, P = 0.0003; *mettl3-14*$^{(M-,Z+)}$, P = 0.0007) in embryos with indicated genotypes at the 0–1-hour, 2–3-hour,

and 5–6-hour stages. The two-sided Student's t-test was used to analyze statistical variance. Data expressed as means of three independent experiments. Error bars indicate mean ± SD. *P < 0.05, **P < 0.01, ***P < 0.001, n.s., not significant. **g**, q-RT-PCR analysis of indicated mRNAs immunoprecipitated by anti-FMR1 antibody. Data expressed as means of three independent experiments. Error bars indicate mean ± SD. **h-j**, q-RT-PCR assays showing expression levels of *Crag* (**h**) (2-3h: $fmr1^{(M-,Z+)}$, P = 0.3; $mettl3\text{-}14^{(M-,Z+)}$, P = 0.36. 5-6h: $fmr1^{(M-,Z+)}$, P = 0.00014; $mettl3\text{-}14^{(M-,Z+)}$, P = 0.0004), *Gdh* (**i**) (2-3h: $fmr1^{(M-,Z+)}$, P = 0.95; $mettl3\text{-}14^{(M-,Z+)}$, P = 0.27. 5-6h: $fmr1^{(M-,Z+)}$, P = 0.0017; $mettl3\text{-}14^{(M-,Z+)}$, P = 8.4e-05) and *Dah* (**j**) (2-3h: $fmr1^{(M-,Z+)}$, P = 0.6; $mettl3\text{-}14^{(M-,Z+)}$, P = 0.016. 5-6h: $fmr1^{(M-,Z+)}$, P = 0.0058; $mettl3\text{-}14^{(M-,Z+)}$, P = 0.0021) in embryos with indicated genotypes at the 0–1-hour, 2–3-hour, and 5–6-hour stages. The two-sided Student's t-test was used to analyze statistical variance. Data expressed as means of 3 independent experiments. Error bars indicate mean ± SD. *P < 0.05, **P < 0.01, ***P < 0.001, n.s., not significant. **k**, PAT assays showing changes in poly(A)-tail length for the indicated transcripts in the 0–1-hour and 5–6-hour stage embryos with indicated genotypes. Representative figures of three independent replicates are shown. Source data are provided as a Source Data file.

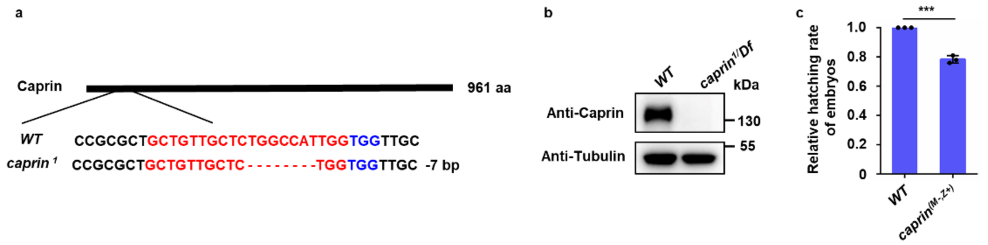

Figure S12. **The loss of maternal Caprin led to relatively weak phenotypes. a**, Schematic representation of *caprin* mutant allele generation using the CRISPR/Cas9 system. The primer sequences of sgRNAs and information for *caprin*^{1} were indicated. **b**, Western blot assays showed that the Caprin protein was completely abolished in *caprin* mutant flies. Representative figures of two independent replicates are shown. **c**, Relative hatching rate of embryos with indicated genotypes (P = 0.0001). The two-sided Student's t-test was used to analyze statistical variance. Data expressed as means of three independent experiments. Error bars indicate mean ± SD. ***$P < 0.001$. Source data are provided as a Source Data file.

Supplementary Table 1. Primers for fly preparation and screening

Primer name	Primer sequence 5' - 3'
mettl3 target 1	TAATACGACTCACTATAGGACTCTTTCCGCGCTACAGGTTTTAGAGCTAGAAATAGC
mettl3-screen-F1	CCTGGAGATGGTGAACTT
mettl3-screen-R1	CGCCGTGGGACTTAAACTT
mettl3 target 2	TAATACGACTCACTATAGGAAGCTGGAAAAGCGCAAGAGTTTTAGAGCTAGAAATAGC
mettl3-screen-F2	CGTGTTTACCTGCAATGTG
mettl3-screen-R2	CACCTGTGTGGAGACAAT
mettl14 target 1	TAATACGACTCACTATAGGGCAACGCGAGGAGGAGGAGTTTTAGAGCTAGAAATAGC
mettl14 target 2	TAATACGACTCACTATAGGTTCCCTTCAGGAAGGTCGGTTTTAGAGCTAGAAATAGC
mettl14-screen-F	GCAAGCGTCGTTTGCTTT
mettl14-screen-R	CAGCGTCTTGACATCTAG
ythdf target 1	TAATACGACTCACTATAGGATGGAGTCAGCAAGTCGATGGTTTTAGAGCTAGAAATAGC
ythdf-screen-F1	GTATTAACCTGCGTTGCG
ythdf-screen-R1	CATGGTTGGCGTTCGCCT
ythdf target 2	TAATACGACTCACTATAGGCGCTCCCAAGAAGACAACCTGTTTTAGAGCTAGAAATAGC
ythdf-screen-F2	TCCTTGGAATTCCAGGAAGC
ythdf-screen-R2	GTGCCGTGTTGCTCGATATG
ythdc target 1	TAATACGACTCACTATAGGCTGTCGATCCTCGGTATCCGTTTTAGAGCTAGAAATAGC
ythdc target 2	TAATACGACTCACTATAGGAAACCAAGCCTTCCAACTTGTTTTAGAGCTAGAAATAGC
ythdc-screen-F	AGTAGCAAGCGGAAAACCA
ythdc-screen-R	TCATGTAGTCGTAGCCCT
caprin target 1	TAATACGACTCACTATAGGCTGTTGCTCTGGCCATTGGGTTTTAGAGCTAGAAATAGC
caprin-screen-F	ATGCCTTCGGCTGCAAAT
caprin-screen-R	CCGAGAACTTAGGCCAA
gRNA-R	AGCACCGACTCGGTGCCACT
Homology arm #1 of *vasa*-F	TCGCTGAAGCAGGTGGAATTCTTGACTTGACCCCTAACCCA
Homology arm #1 of *vasa*-R	AGACAGTAGCTTCATTGATATTTTTTTTTAATTTGGCTGAAAG
Homology arm #2 of *vasa*-F	GAGTACGGTGGGTAGTTCGTATTGCTTCGTACAAAG
Homology arm #2 of *vasa*-R	GCCGCTAGCATGCAAGAATTCTCGTGCCAAGTTTACCATACT
vasa CRISPR/Cas target 1	TATATAGGAAAGATATCCGGGTGAACTTCGATATCAATATGTCTGACGACGTTTTAGAGCTAGAAATAGCAAG
vasa CRISPR/Cas target 2	ATTTTAACTTGCTATTTCTAGCTCTAAAACCATCATCCCAGTCGTCAGACGACGTTAAATTGAAAATAGGTC

Supplementary Table 2. Primers for RNA probe synthesis

Primer name	Primer sequence 5’ - 3’
RNA probe including “GGACU”, “AGACU” and “GAACU”-F	TAATACGACTCACTATAGGGGACTAGACTGAACTGGGACTAGACTGAACTGGGACTAGACTGAACTGTAC
RNA probe including “GGACU”, “AGACU” and “GAACU”-R	GTACAGTTCAGTCTAGTCCCAGTTCAGTCTAGTCCCAGTTCAGTCTAGTCCCCTATAGTGAGTCGTATTA
RNA probe including “GGCCU”, “AGCCU” and “GACCU”-F (Mutant RNA)	TAATACGACTCACTATAGGGGCCTAGCCTGACCTGGGCCTAGCCTGACCTGGGCCTAGCCTGACCTGT
RNA probe including “GGCCU”, “AGCCU” and “GACCU”-R(Mutant RNA)	ACAGGTCAGGCTAGGCCCAGGTCAGGCTAGGCCCAGGTCAGGCTAGGCCCCTATAGTGAGTCGTATTA
GGACU (9x) RNA probe-F	TAATACGACTCACTATAGGGGGACTGGACTGGACTGGGACTGGACTGGACTGGGACTGGACTGGACTGTAC
GGACU (9x) RNA probe-R	GTACAGTCCAGTCCAGTCCCAGTCCAGTCCAGTCCCAGTCCAGTCCAGTCCCCCTATAGTGAGTCGTATTA
AGACU (9x) RNA probe-F	TAATACGACTCACTATAGGGAGACTAGACTAGACTGAGACTAGACTAGACTGAGACTAGACTAGACTGTAC
AGACU (9x) RNA probe-R	GTACAGTCTAGTCTAGTCTCAGTCTAGTCTAGTCTCAGTCTAGTCTAGTCTCCCTATAGTGAGTCGTATTA
GAACU (9x) RNA probe-F	TAATACGACTCACTATAGGGGAACTGAACTGAACTGGAACTGAACTGAACTGGAACTGAACTGAACTGTAC
GAACU (9x) RNA probe-R	GTACAGTTCAGTTCAGTTCCAGTTCAGTTCAGTTCCAGTTCAGTTCAGTTCCCCTATAGTGAGTCGTATTA
AAACU (9x) RNA probe-F	TAATACGACTCACTATAGGGAAACTAAACTAAACTGAAACTAAACTAAACTGAAACTAAACTAAACTGTAC
AAACU (9x) RNA probe-R	GTACAGTTTAGTTTAGTTTCAGTTTAGTTTAGTTTCAGTTTAGTTTAGTTTCCCTATAGTGAGTCGTATTA
AGCCU (9x) RNA probe-F	TAATACGACTCACTATAGGGAGCCTAGCCTAGCCTGAGCCTAGCCTAGCCTGAGCCTAGCCTAGCCTGTAC
AGCCU (9x) RNA probe-R	GTACAGGCTAGGCTAGGCTCAGGCTAGGCTAGGCTCAGGCTAGGCTAGGCTCCCTATAGTGAGTCGTATTA
AGACA (9x) RNA probe-F	TAATACGACTCACTATAGGTAGACAAGACAAGACAAGACAAGACAAGACAAGACAAGACAAGACAGTACT
AGACA (9x) RNA probe-R	AGTACTGTCTTGTCTTGTCTTGTCTTGTCTTGTCTTGTCTTGTCTTGTCTACCTATAGTGAGTCGTATTA
AGACC (9x) RNA probe-F	TAATACGACTCACTATAGGTAGACCAGACCAGACCAGACCAGACCAGACCAGACCAGACCAGACCGTACT
AGACC (9x) RNA probe-R	AGTACGGTCTGGTCTGGTCTGGTCTGGTCTGGTCTGGTCTGGTCTGGTCTACCTATAGTGAGTCGTATTA

Supplementary Table 3. Primers for q-RT-PCR and PAT assay

Primer name	Primer sequence 5' - 3'
RASSF8 q-RT-PCR-F	CATCAACAGCAGACCAATAGG
RASSF8 q-RT-PCR-R	GCAAATTCACCTCCTTCATCTC
hdc q-RT-PCR-F	GGAGAACGTGAATTCTTCAG
hdc q-RT-PCR-R	CTCGTTCAGGTGTATCAGG
HIP q-RT-PCR-F	CGGAAATATGTCTGATATACTGGG
HIP q-RT-PCR-R	GGGCACAATCTTCTTGATAAGG
Crag q-RT-PCR-F	GACTCTAACCTGAACACCAC
Crag q-RT-PCR-R	ACCAGCGGATTTAGATACGG
Gdh q-RT-PCR-F	GATTGCCGATACTTATGCCA
Gdh q-RT-PCR-R	CCTCGTTGATGAAGTTCTCC
Dah q-RT-PCR-F	ACAAATCGGATTACTCGCTC
Dah q-RT-PCR-R	GCTTAAAGTTCACCATCTCGG
Gcl q-RT-PCR-F	CTACGAACCCAGTACCTGAC
Gcl q-RT-PCR-R	CAAAGAACTGCTCATCGTCC
Actin 5C q-RT-PCR-F	CTGATGAAGATCCTGACCGA
Actin 5C q-RT-PCR-R	ATGATGGAGTTGTAGGTGGT
RASSF8 PAT assay-F	CACAAACTGATGATGATAGTG
hdc PAT assay-F	TACGAGCCAACTCTCCTGAA
HIP PAT assay-F	GCAAATGCATTTAGCCCCCCA
Gdh PAT assay-F	AGTTGAACAAGTTGCGAGCT
Carg PAT assay-F	CGAACTTTCCTAAGTGAAAGC
Dah PAT assay-F	CTTACAACTCTGACAAGAATGC
Gcl PAT assay-F	ACGCGGCAAAGAAATTATGG
β tub56D PAT assay-F	GCTGAGGTCGACGAGAACTAA
