# Supplementary material for: Dynamic FMR1 granule phase switch instructed by m6A modification contributes to maternal RNA decay
Source: Nat Commun. 2022 Feb 14;13:859. doi: 10.1038/s41467-022-28547-7 (PMC8844045; doi:10.1038/s41467-022-28547-7)
Supplement: Supplementary file 3 — Description of Additional Supplementary Files [file 41467_2022_28547_MOESM3_ESM.pdf]

Description of Additional Supplementary Files

File Name: Supplementary Data 1
Description: Expression profile of clustered gene groups by RNA sequencing. Table contains degraded maternal mRNAs, stable maternal mRNAs and zygotically expressed mRNAs.

File Name: Supplementary Data 2
Description: Differential gene expression analysis. Table lists differentially expressed genes in *mettl3-14*$^{(M-,Z+)}$ and *fmr1*$^{(M-,Z+)}$.

File Name: Supplementary Data 3
Description: The m6A datasets of embryos at the 0–1-hour and 5–6-hour stages.

File Name: Supplementary Data 4
Description: Proteomics Data. Table lists the mass spectrometry proteomics data generated in this study.
