# Supplementary material for: Dynamic FMR1 granule phase switch instructed by m6A modification contributes to maternal RNA decay
Source: Nat Commun. 2022 Feb 14;13:859. doi: 10.1038/s41467-022-28547-7 (PMC8844045; doi:10.1038/s41467-022-28547-7)
Supplement: Supplementary file 8 — Reporting Summary [file 41467_2022_28547_MOESM8_ESM.pdf]

nature portfolio

Corresponding author(s): Dahua Chen

Last updated by author(s): Dec 21, 2021

Reporting Summary

Nature Portfolio wishes to improve the reproducibility of the work that we publish. This form provides structure for consistency and transparency in reporting. For further information on Nature Portfolio policies, see our Editorial Policies and the Editorial Policy Checklist.

Statistics

For all statistical analyses, confirm that the following items are present in the figure legend, table legend, main text, or Methods section.

n/a	Confirmed	
☐	☒	The exact sample size (*n*) for each experimental group/condition, given as a discrete number and unit of measurement
☐	☒	A statement on whether measurements were taken from distinct samples or whether the same sample was measured repeatedly
☐	☒	The statistical test(s) used AND whether they are one- or two-sided<br>*Only common tests should be described solely by name; describe more complex techniques in the Methods section.*
☐	☒	A description of all covariates tested
☐	☒	A description of any assumptions or corrections, such as tests of normality and adjustment for multiple comparisons
☐	☒	A full description of the statistical parameters including central tendency (e.g. means) or other basic estimates (e.g. regression coefficient) AND variation (e.g. standard deviation) or associated estimates of uncertainty (e.g. confidence intervals)
☐	☒	For null hypothesis testing, the test statistic (e.g. *F*, *t*, *r*) with confidence intervals, effect sizes, degrees of freedom and *P* value noted<br>*Give P values as exact values whenever suitable.*
☒	☐	For Bayesian analysis, information on the choice of priors and Markov chain Monte Carlo settings
☒	☐	For hierarchical and complex designs, identification of the appropriate level for tests and full reporting of outcomes
☐	☒	Estimates of effect sizes (e.g. Cohen's *d*, Pearson's *r*), indicating how they were calculated

Our web collection on statistics for biologists contains articles on many of the points above.

Software and code

Policy information about availability of computer code

Data collection	The images were collected on a Zeiss LSM 710 Meta confocal microscopy or a Nikon Eclipse TI microscopy. The LC-MS/MS data was collected on a LTQ orbitrap elite and orbitrap exploris 480. The SPR data was collected on Biacore 8K instrument. The quantitation of band intensity was measured using ImageJ software (1.46r). No custom codes were developed in the study.
Data analysis	The granule size was calculated with the analyze particle tools in Image J 1.46r. Fluorescence intensity along the straight line of FMR1 and pAbp proteins was calculated with the plot profile tool in Image J 1.46r. The resulting LC-MS/MS data were processed using MaxQuant (v1.6.1.0) and Skyline software (Skyline 21. 1. 0. 146). Equilibrium and kinetic constants were calculated by a global fit to 1:1 Langmuir binding model (Biacore 8K evaluation software).The FastQC software was used to confirm the high quality of the sequencing data. The clean reads RNA-seq were mapped to the D. melanogaster genome with TopHat version 2.0. The FPKM value for gene expression and differential expression analysis was carried out by cuffdiff from the cufflinks package (version 2.21). For MeRIP-m6A-Seq data analysis, adaptor-trimming and low quality reads filtering were performed by the Cutadapt software (version 1.12), the remaining reads were aligned to Drosophila reference genome by HISAT2 (version 2.1.0). The peak calling analysis was performed by MACS2 (version 2.2.1). The two replicates of m6A peaks were intersected with each other by Bedtools package (version 2.25.0). Differentially methylated sites on transcripts were identified by diffReps (version 1.55.3). The m6A changes were normalized by using ngs.plot software (v2.61).

For manuscripts utilizing custom algorithms or software that are central to the research but not yet described in published literature, software must be made available to editors and reviewers. We strongly encourage code deposition in a community repository (e.g. GitHub). See the Nature Portfolio guidelines for submitting code & software for further information.

Data

Policy information about availability of data

All manuscripts must include a data availability statement. This statement should provide the following information, where applicable:

- Accession codes, unique identifiers, or web links for publicly available datasets
- A description of any restrictions on data availability
- For clinical datasets or third party data, please ensure that the statement adheres to our policy

The raw sequencing data generated in this study have been deposited in the NCBI's Gene Expression Omnibus and are accessible through GEO Series accession number GSE143821[https://www.ncbi.nlm.nih.gov/geo/query/acc.cgi?acc=GSE143821]. The mass spectrometry proteomics data generated in this study have been deposited to the ProteomeXchange Consortium via the iProX partner repository with the dataset identifier PXD026356 [http://proteomecentral.proteomexchange.org/cgi/GetDataset?ID=PXD026356].

Field-specific reporting

Please select the one below that is the best fit for your research. If you are not sure, read the appropriate sections before making your selection.

☒ Life sciences ☐ Behavioural & social sciences ☐ Ecological, evolutionary & environmental sciences

For a reference copy of the document with all sections, see nature.com/documents/nr-reporting-summary-flat.pdf

Life sciences study design

All studies must disclose on these points even when the disclosure is negative.

Sample size	Sample sizes used in our experiments were determined according to our experience as well as published literatures.
Data exclusions	No data were excluded from analysis.
Replication	Genetic experiments, q-RT-PCR and MS were carried out three times. RNA-Seq and MeRIP-m6A-Seq analysis were performed with two biological replicates for each experiment. All attempts at replication were successful.
Randomization	Flies were randomly assigned to experimental groups.
Blinding	The investigators were not blinded to during experiments and outcome assessment, because the drosophila were maintained in the same way.

Reporting for specific materials, systems and methods

We require information from authors about some types of materials, experimental systems and methods used in many studies. Here, indicate whether each material, system or method listed is relevant to your study. If you are not sure if a list item applies to your research, read the appropriate section before selecting a response.

Materials & experimental systems

n/a	Involved in the study	
☐	☒	Antibodies
☒	☐	Eukaryotic cell lines
☒	☐	Palaeontology and archaeology
☐	☒	Animals and other organisms
☒	☐	Human research participants
☒	☐	Clinical data
☒	☐	Dual use research of concern

Methods

n/a	Involved in the study	
☒	☐	ChIP-seq
☒	☐	Flow cytometry
☒	☐	MRI-based neuroimaging

Antibodies

Antibodies used

Rabbit polyclonal anti-N6-methyladenosine (m6A), Millipore, IP: 1:100, ABE572
Mouse monoclonal anti-Drosophila FMR1, Abcam, IP: 1:100, WB: 1:2000, IHC: 1:2000, ab10299
Rabbit polyclonal anti-Myc, MBL, WB:1:2000, 562
Mouse monoclonal anti-β-Tubulin, CWBIO, WB: 1:2000, CW0098M
Mouse polyclonal anti-Mettl3, WB: 1:1000, This paper
Mouse polyclonal anti-Mettl14, WB: 1:1000, This paper
Mouse polyclonal anti-Ythdf, WB: 1:1000, This paper
Mouse polyclonal anti-Ythdc, WB: 1:1000, This paper

	Rabbit polyclonal anti-pAbp, WB:1:2000, IHC:1:2000, This paper<br>Rabbit polyclonal anti-Caprin, WB:1:2000, This paper<br>Streptavidin (HRP), Abcam, EMSA:1:5000, ab7403<br>HRP-goat anti-mouse IgG (H+L), KPL, WB:1:5000, 074-1806<br>HRP-goat anti-rabbit IgG (H+L), KPL, WB:1:5000, 074-1506<br>Alexa Fluor 555 goat anti-mouse IgG (H+), invitrogen, IHC:1:2000, A21422<br>Alexa Fluor 488 goat anti-rabbit IgG (H+), invitrogen, IHC:1:2000, A11034
Validation	The commercial antibodies were validated based on the information from the manufacturer's instrutions and were supported by multiple publications. Antibodies that were made in this paper were validated by performing western blot using null mutants as control.

Animals and other organisms

Policy information about studies involving animals; ARRIVE guidelines recommended for reporting animal research

Laboratory animals	Drosophila melanogaster: w1118, fmr1Δ50, fmr1Δ113, mettl3 1, mettl3 2, mettl14 1, mettl14 2, ythdf1, ythdf2, ythdc 1, ythdc 2, P{uasp-myc-fmr1}, P{uasp-myc-fmr1ΔLC}, P{uasp-myc-fmr1V311K}, P{vasa-gal4:vp16}, P{uasp-gfp-fmr1}, P{uasp-gfp-fmr1ΔLC} and P{uasp-gfp-fmr1 KH2-GDDG}. 3-5 day old female were used to collect embryos.
Wild animals	The study did not involve wild animals
Field-collected samples	The study did not involve field collected samples.
Ethics oversight	No approval required for Drosophila melanogaster. The lab is licensed to handle Drosophila.

Note that full information on the approval of the study protocol must also be provided in the manuscript.
