# Supplementary material for: Dynamic FMR1 granule phase switch instructed by m6A modification contributes to maternal RNA decay
Source: Nat Commun. 2022 Feb 14;13:859. doi: 10.1038/s41467-022-28547-7 (PMC8844045; doi:10.1038/s41467-022-28547-7)
Supplement: Supplementary file 9 — Source data [file 41467_2022_28547_MOESM9_ESM.zip › Source data/uncropped blots.pdf]

Figure 1f

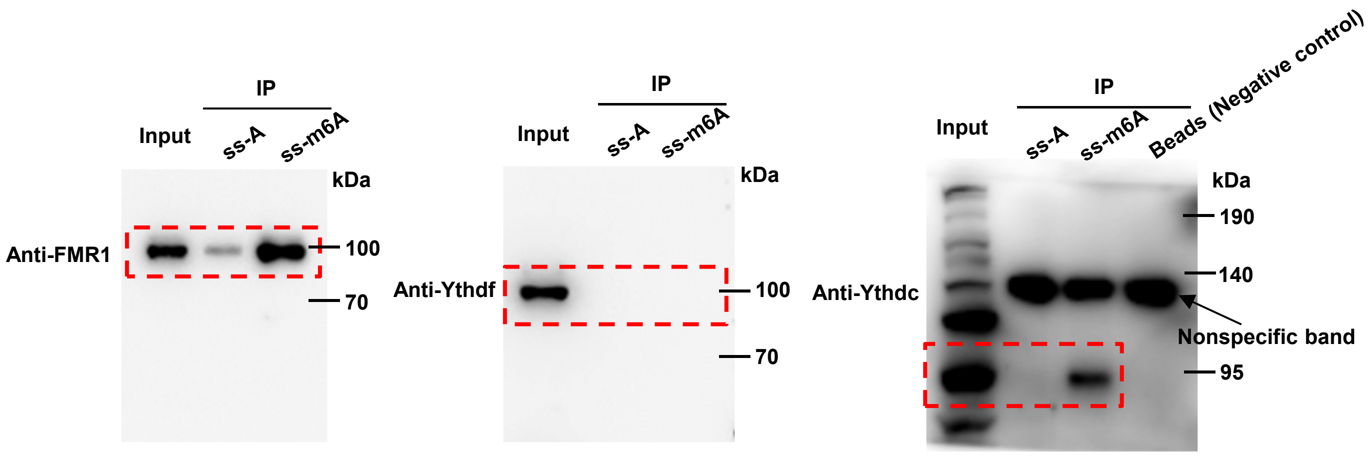

Figure 1g

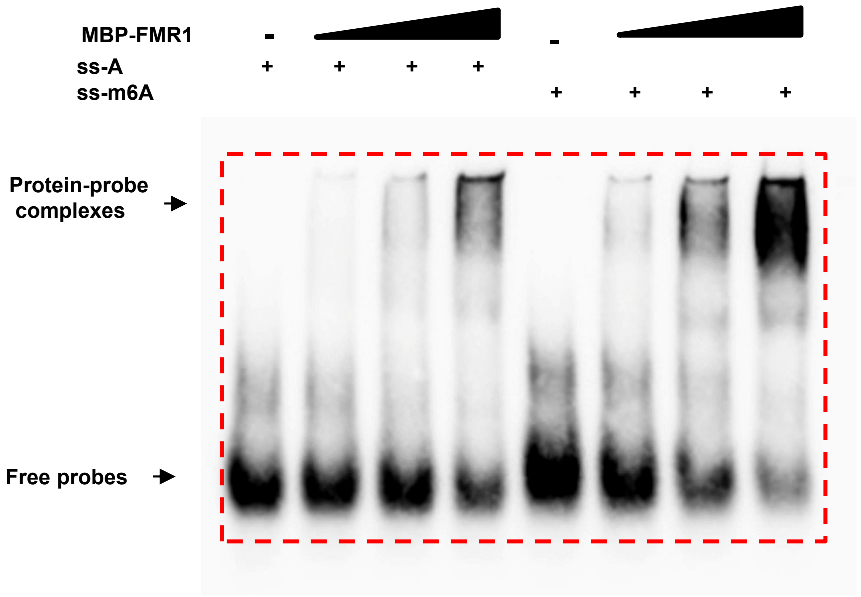

Figure 2b

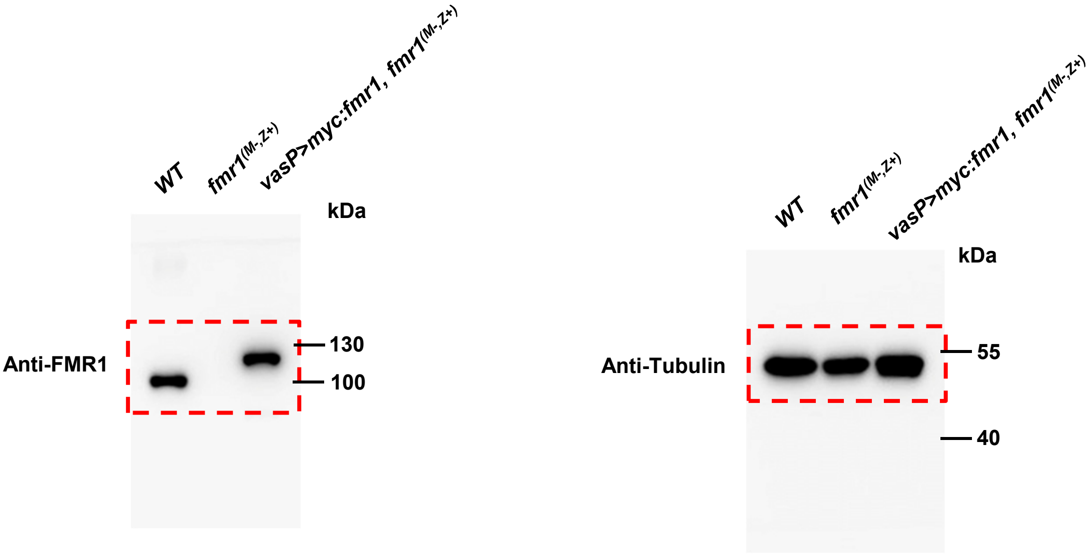

Figure 2e

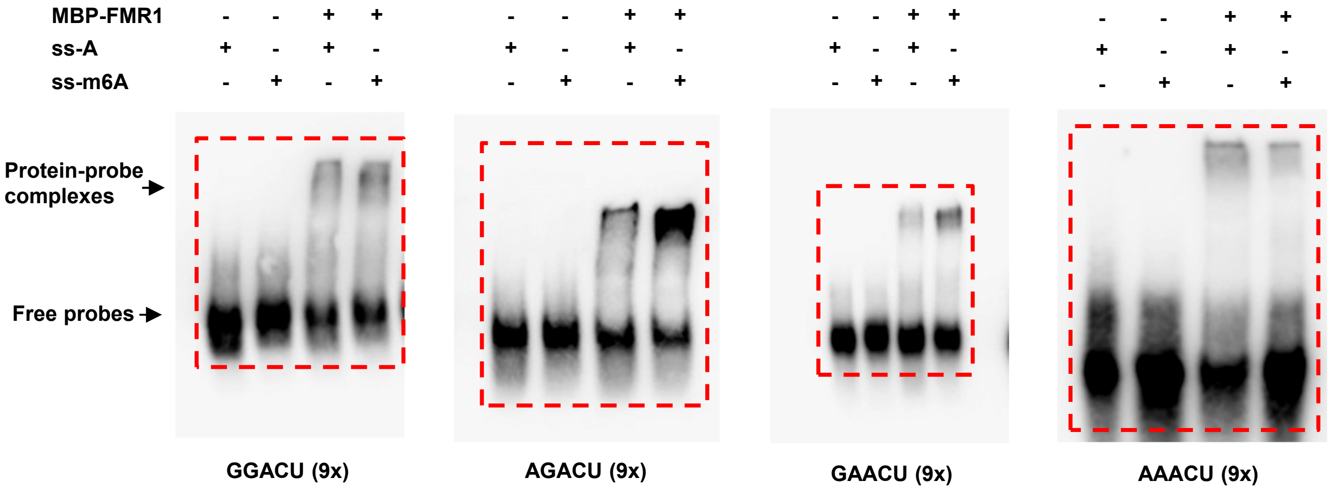

Figure 2g

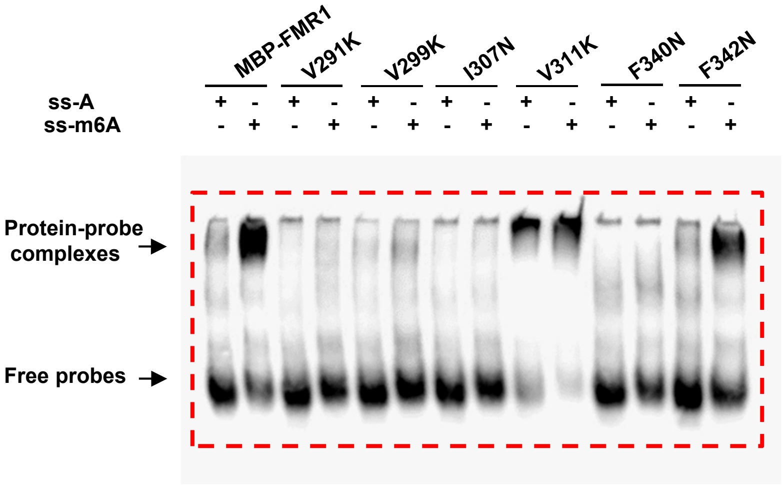

Figure 3b

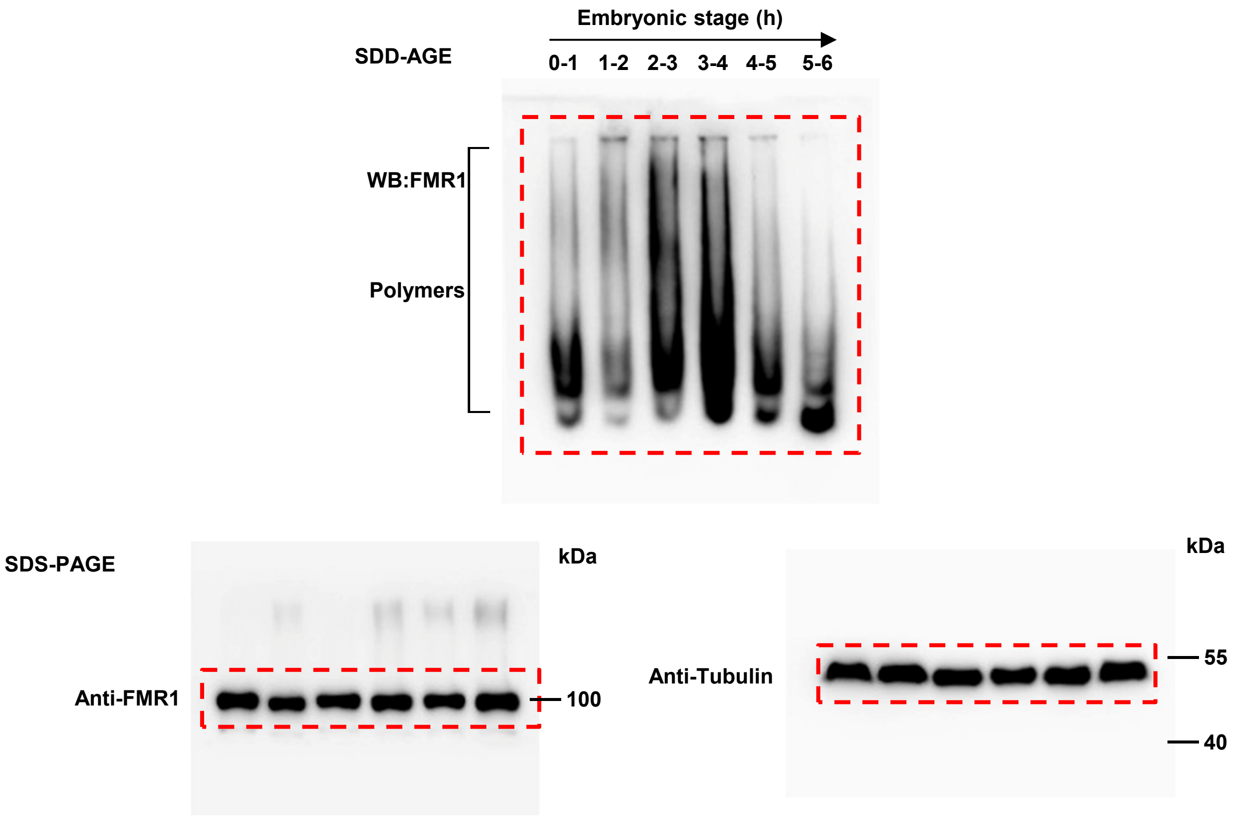

Figure 3f

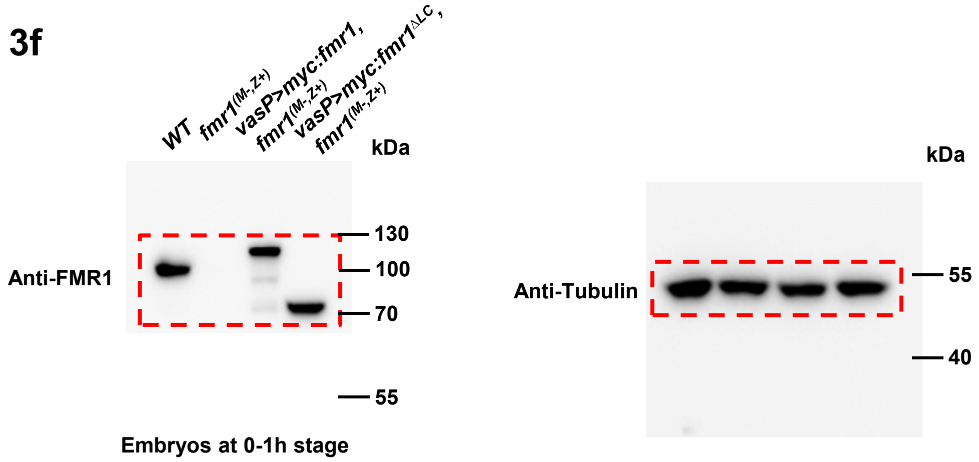

Figure 3g

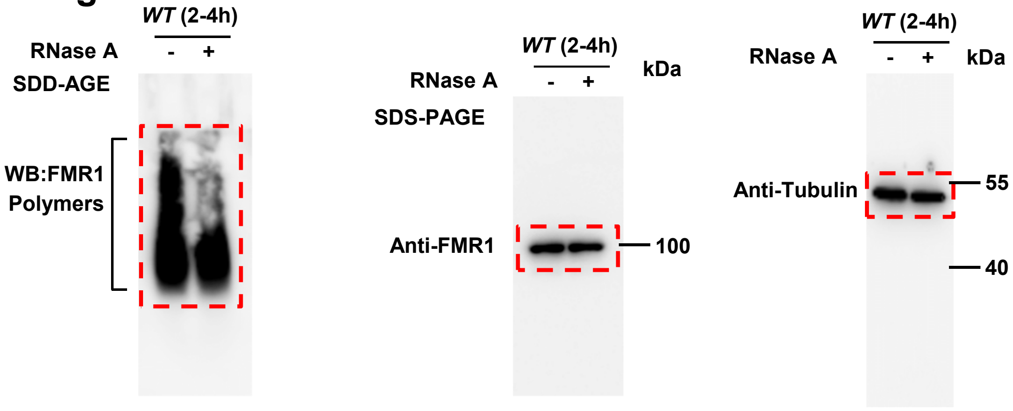

Figure 5e

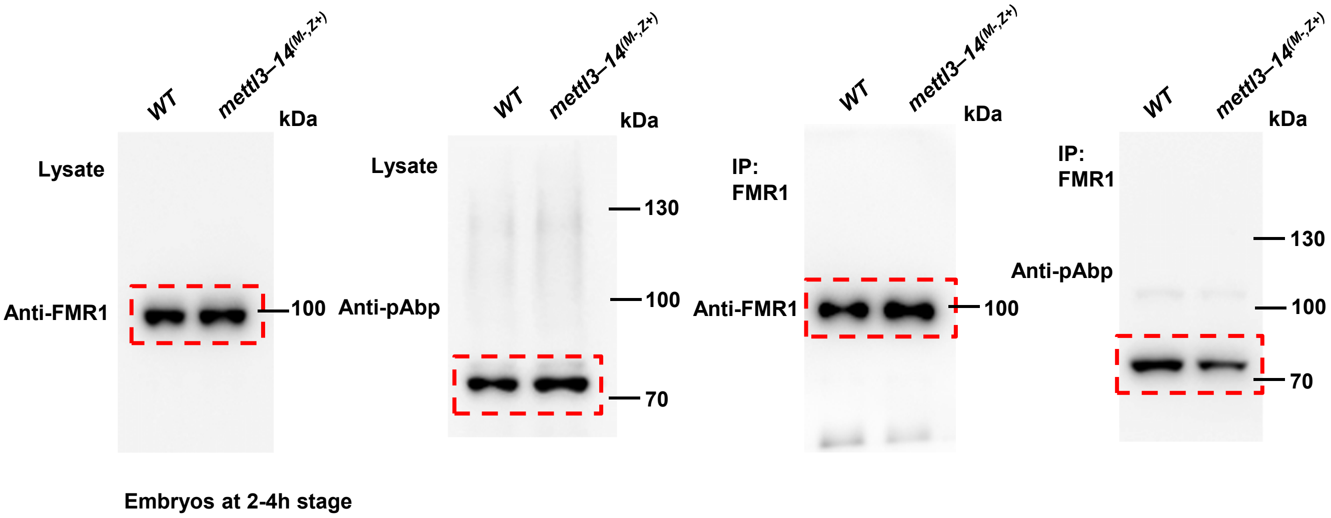

Figure 5f

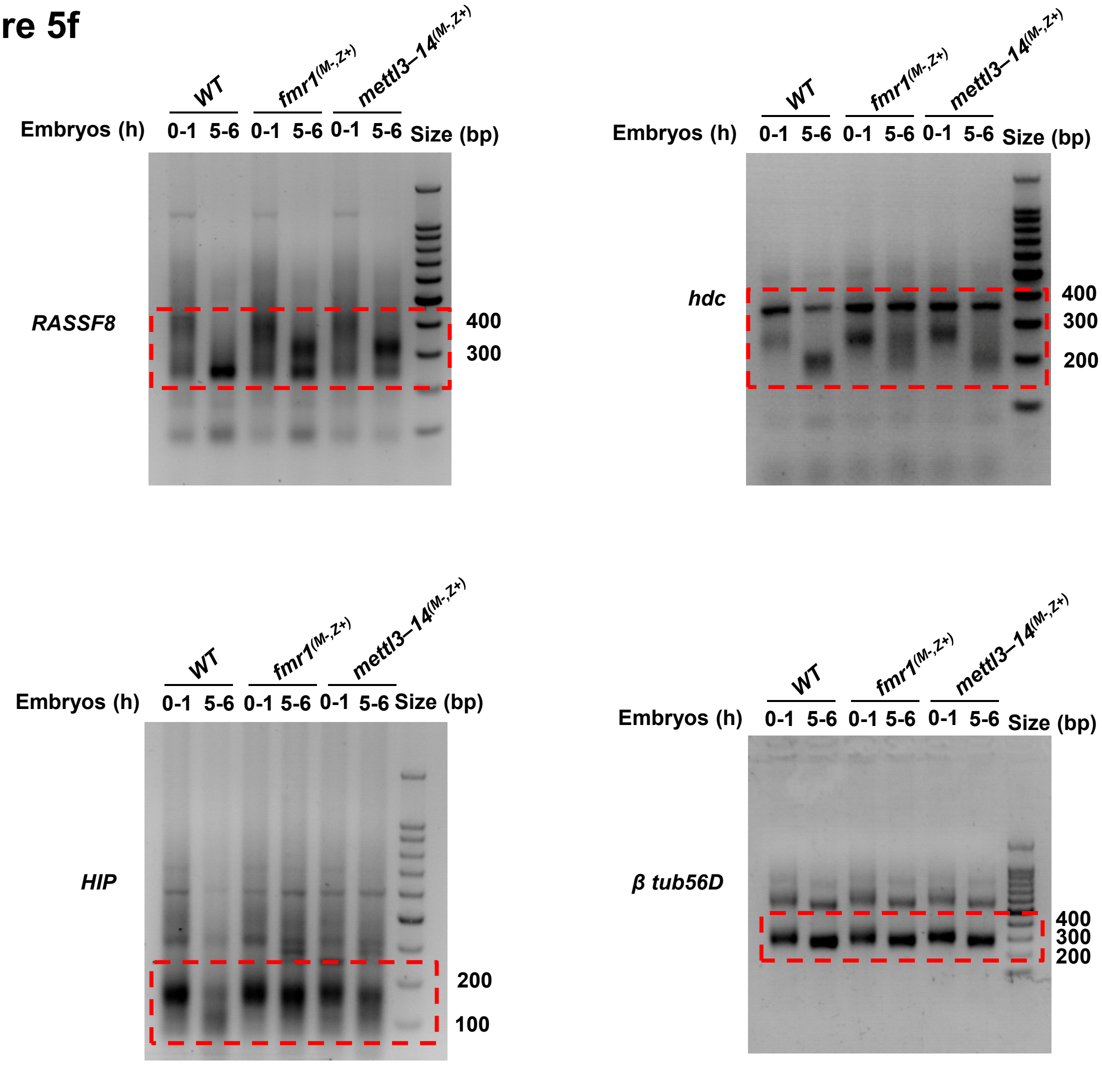

Figure S1d

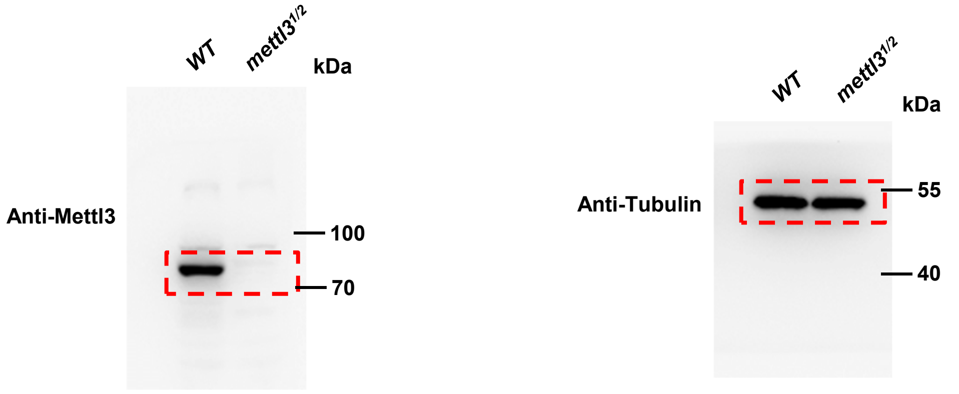

Figure S1f

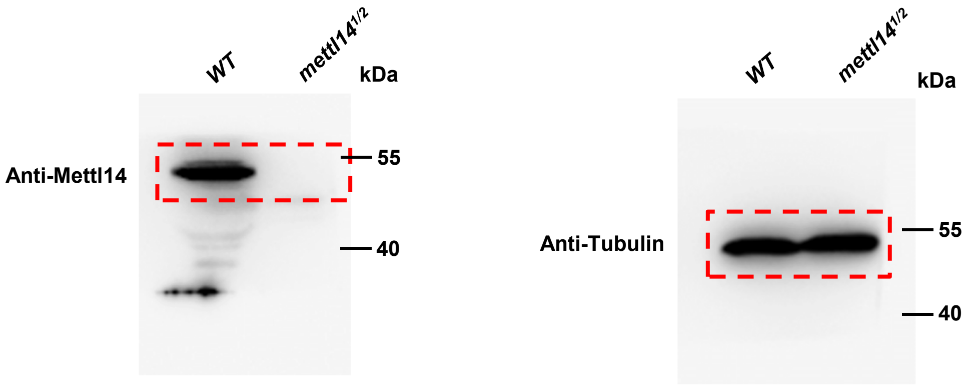

Figure S2b

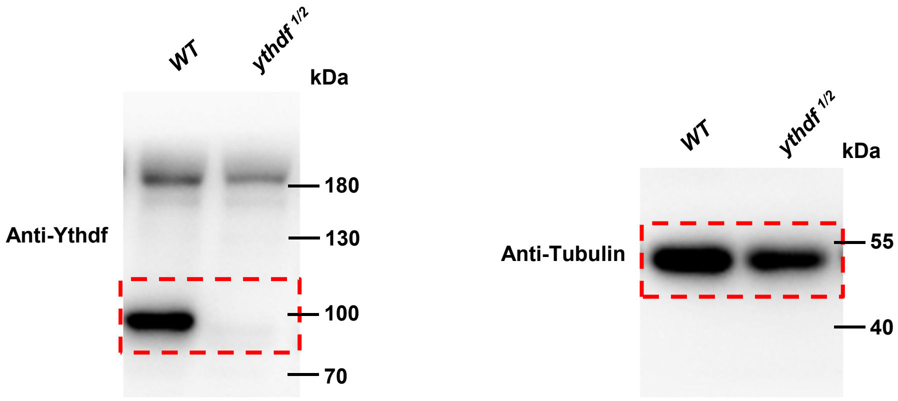

Figure S2d

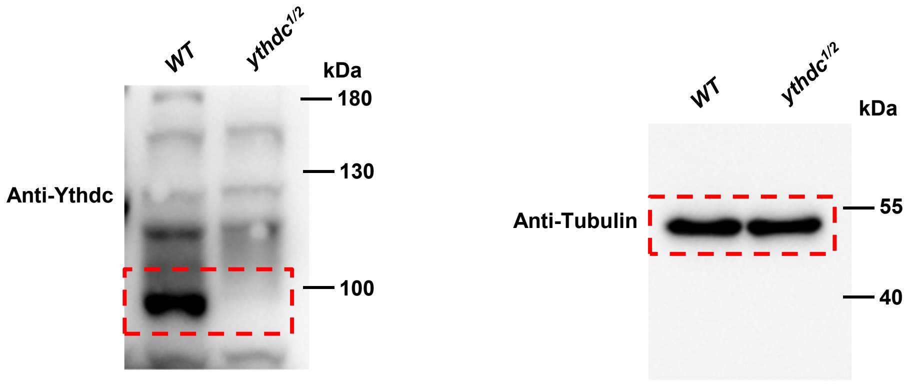

Figure S3b

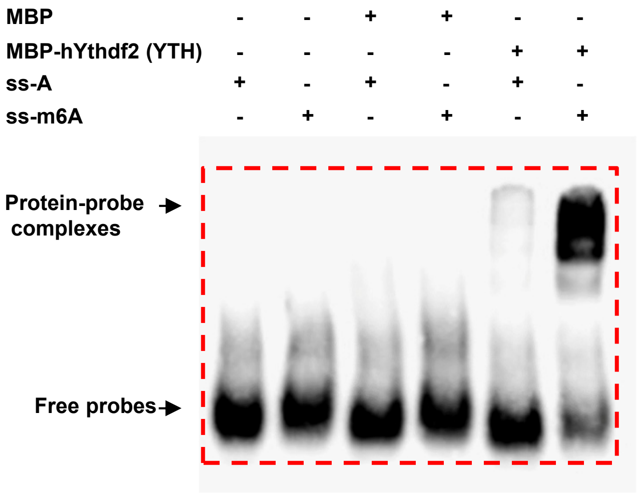

Figure S3c

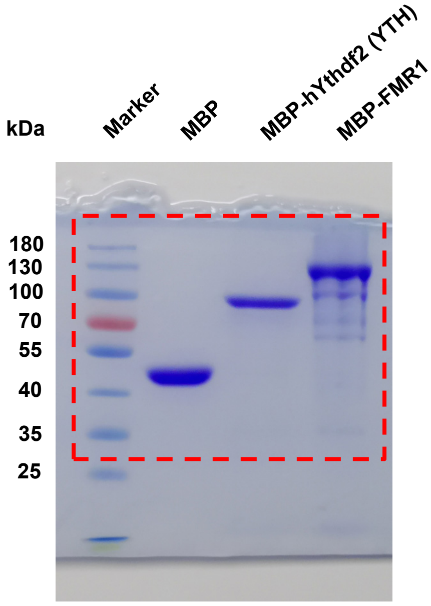

Figure S3e

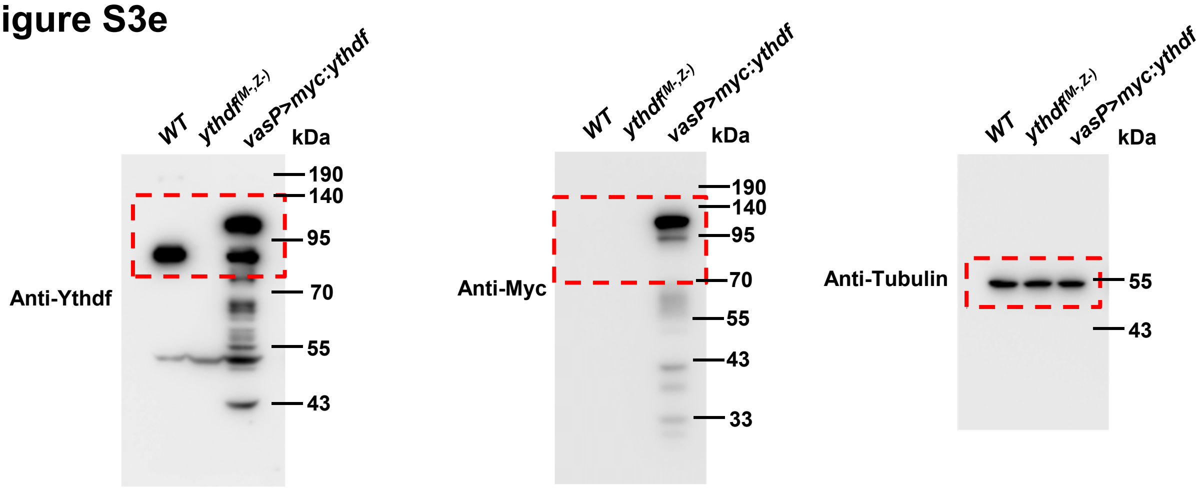

Figure S4a

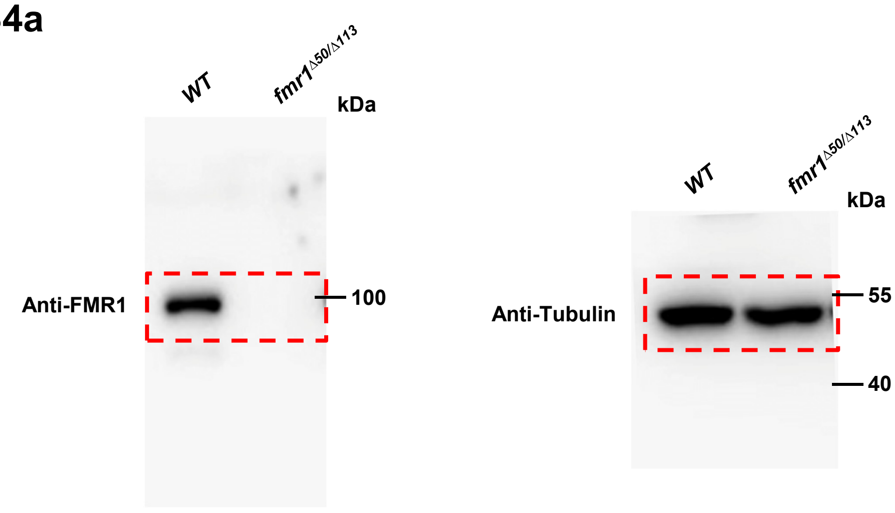

Figure S4g

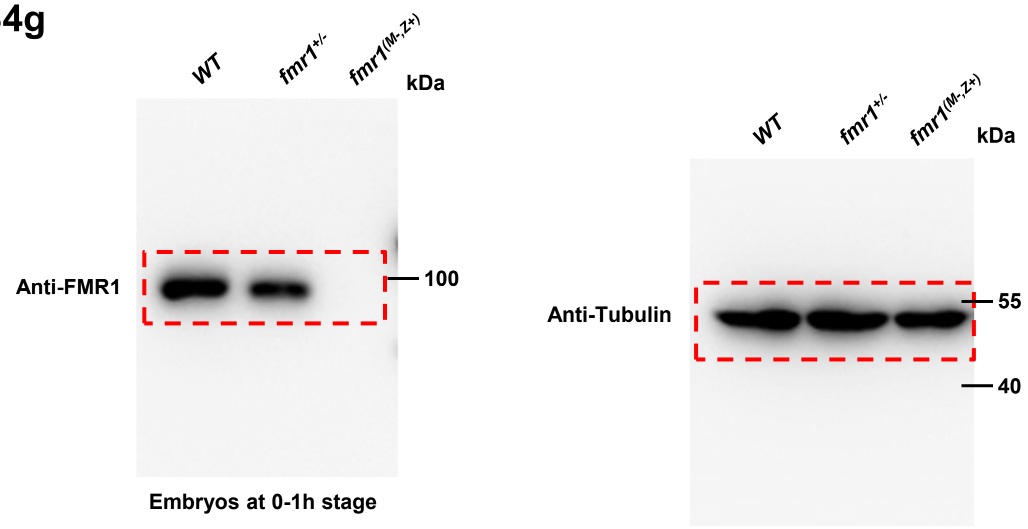

Figure S5a

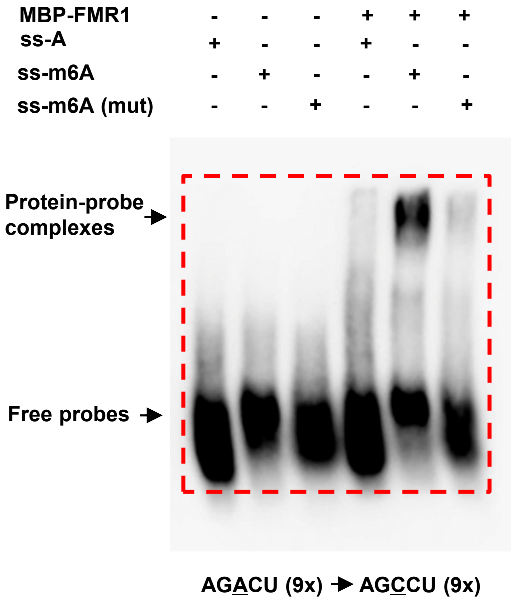

Figure S5b

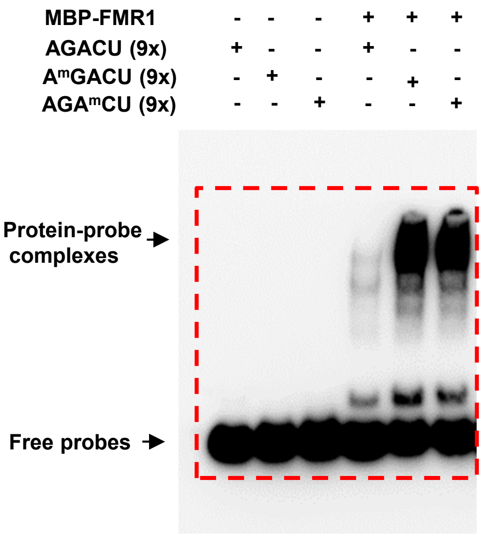

Figure S5c

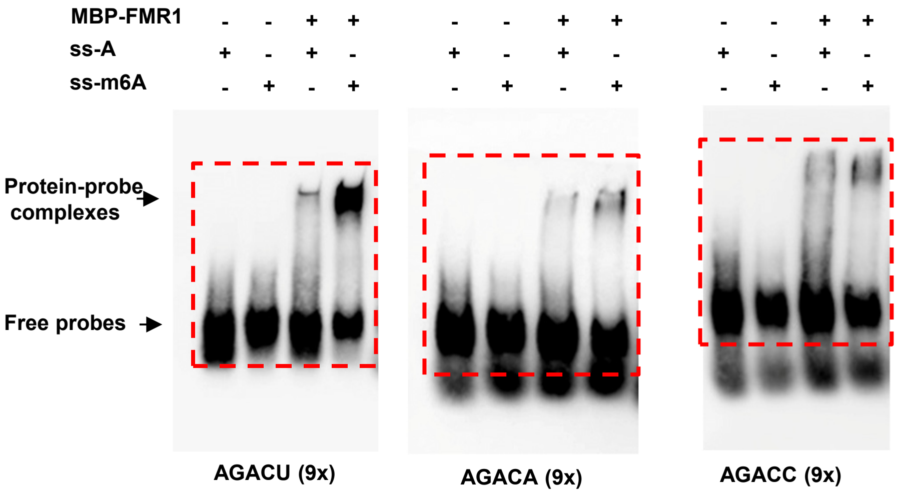

Figure S6b

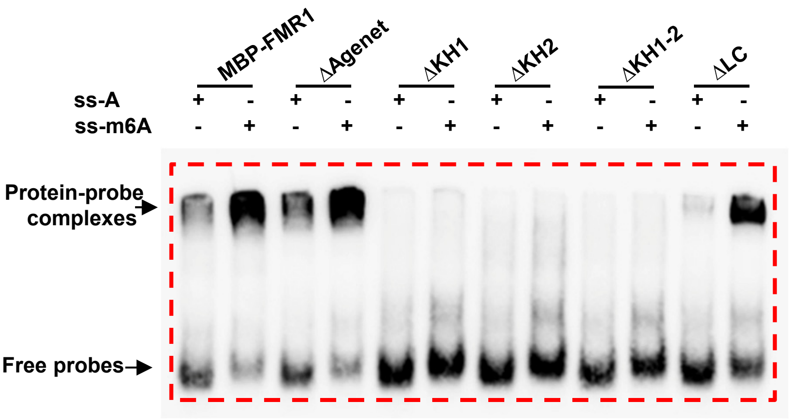

Figure S6c

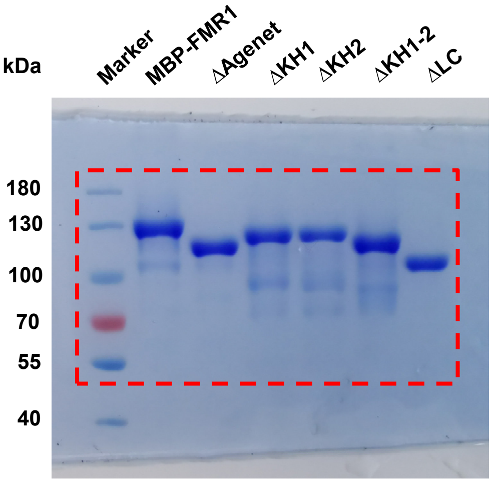

Figure S6d

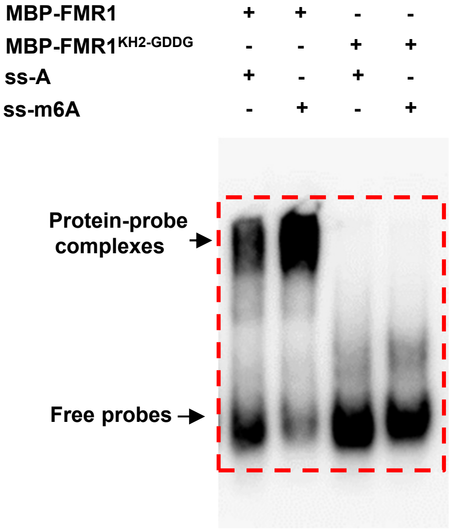

Figure S7c

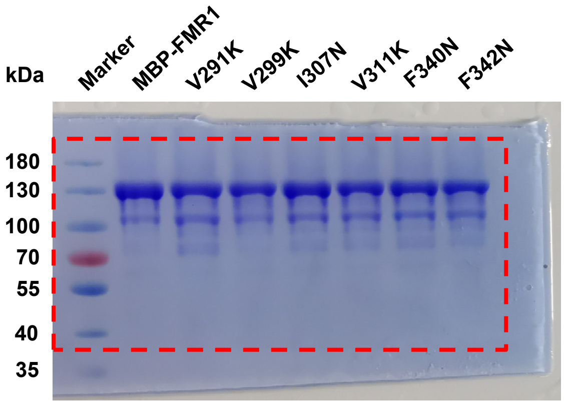

Figure S7e

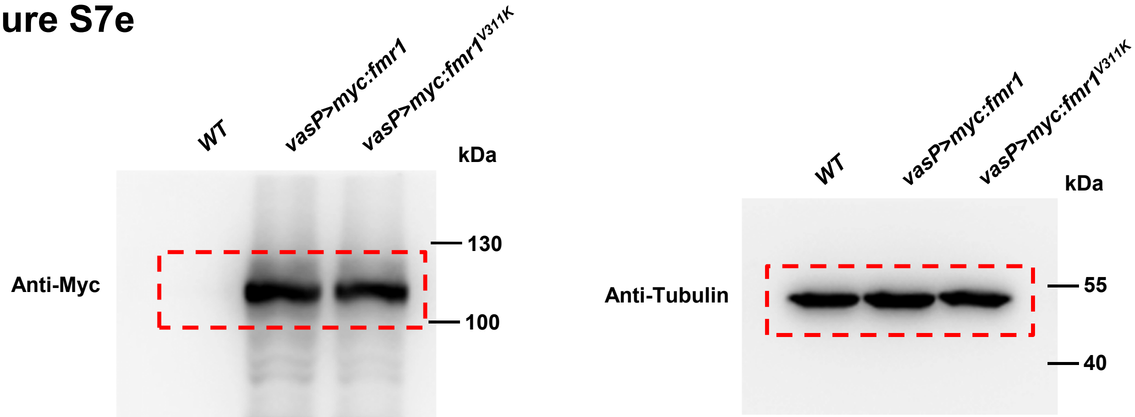

Embryos at 0-1h stage

Figure S7f

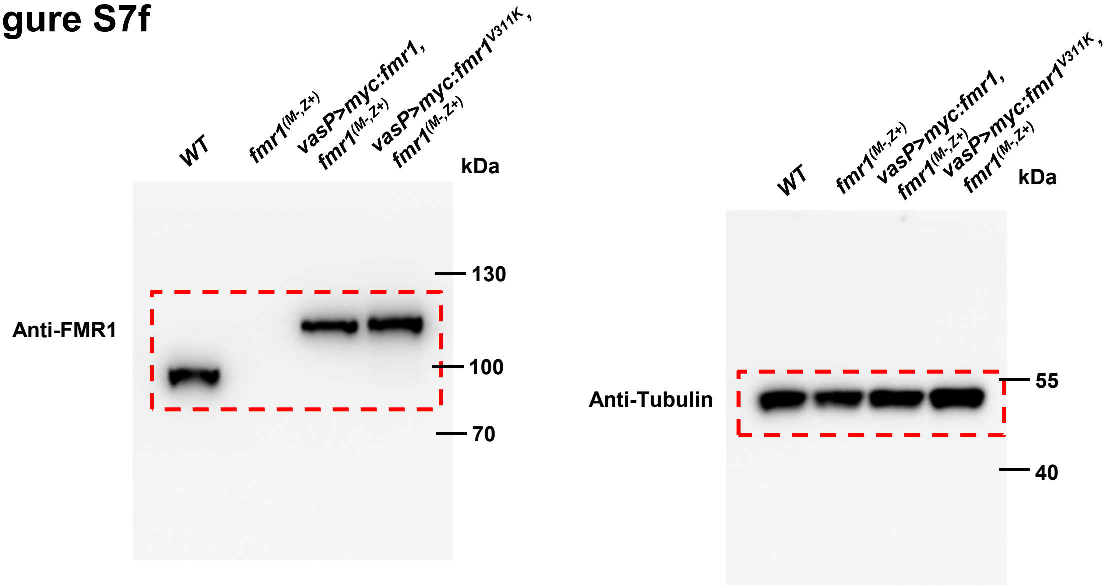

Embryos at 0-1h stage

Figure S9a

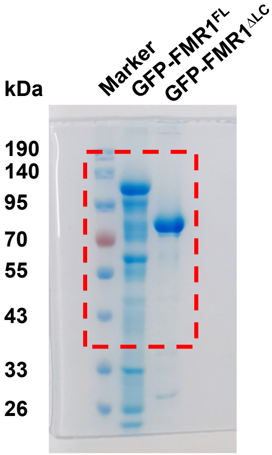

Figure S9f

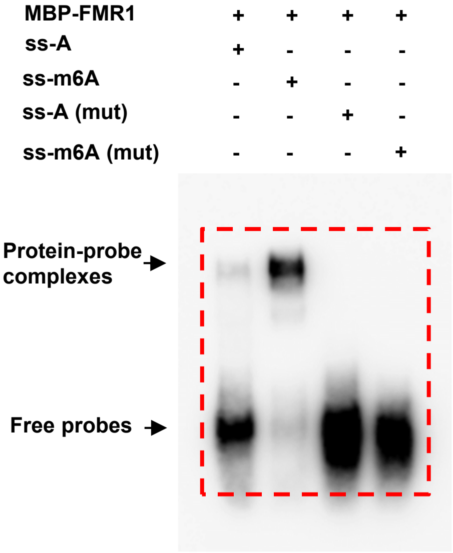

Figure S9i

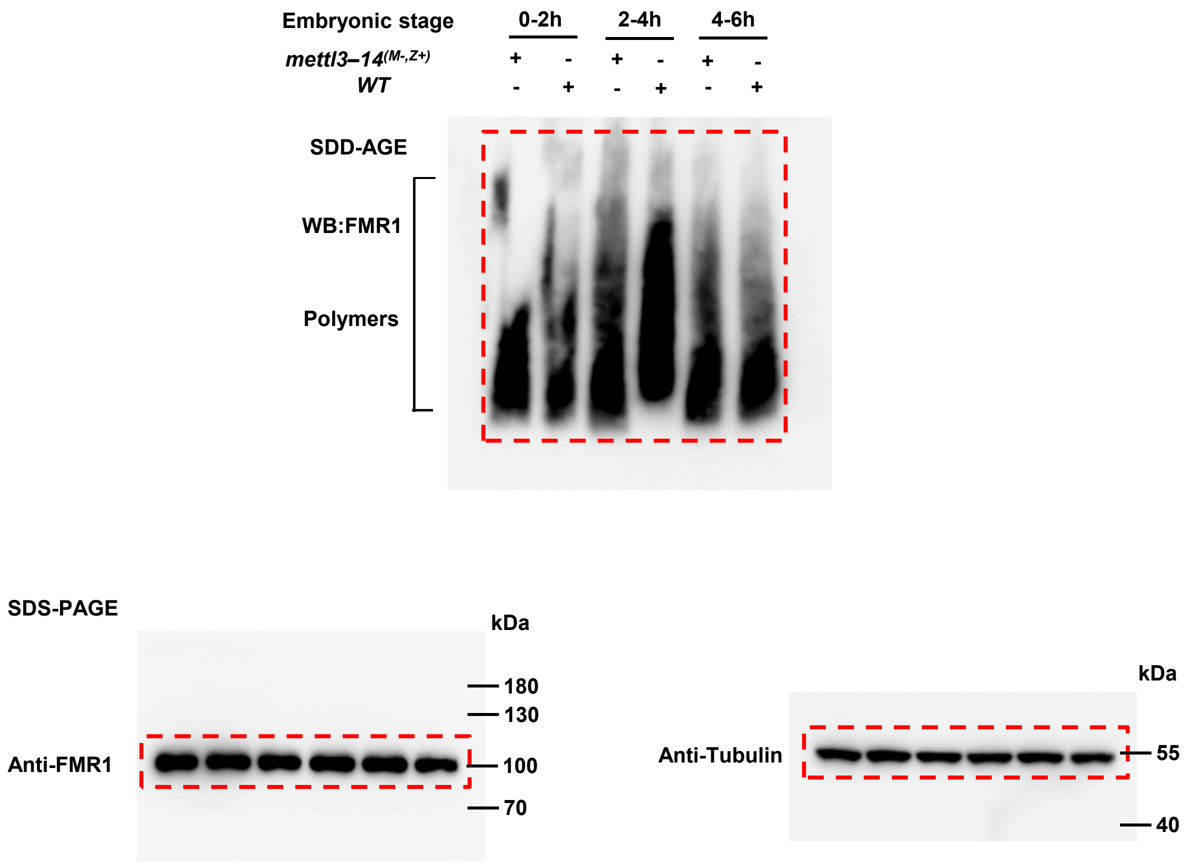

Figure S10e

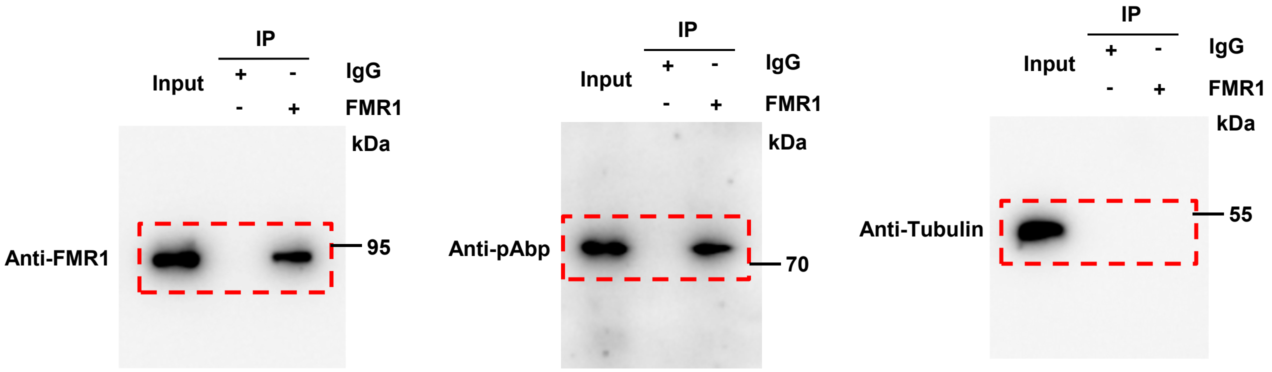

Figure S11k

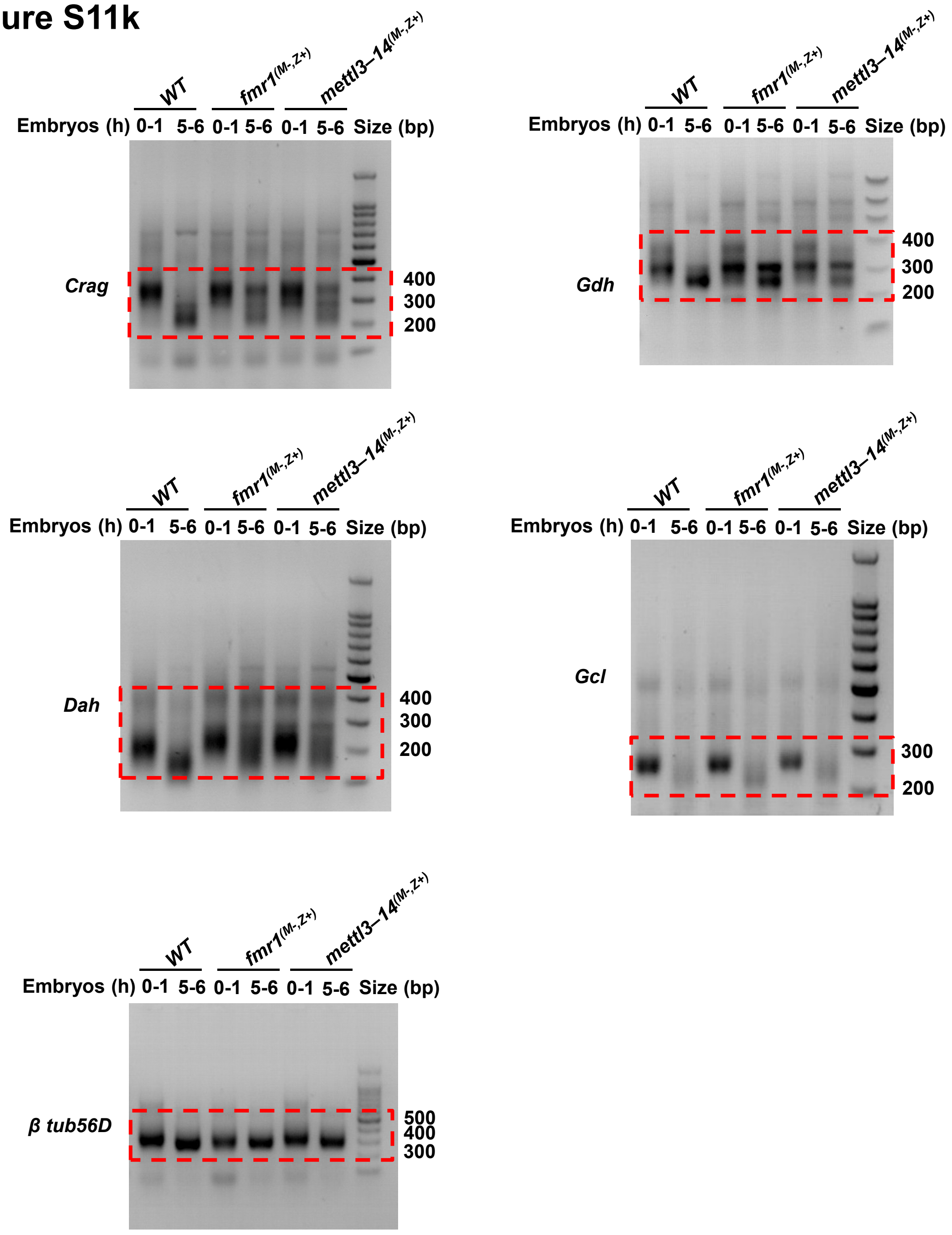
WT
fmr1(M-,Z+)
mettl3–14(M-,Z+)
Embryos (h) 0-1 5-6 0-1 5-6 0-1 5-6 Size (bp)
Crag
400
300
200
Gdh
400
300
200
Dah
400
300
200
Gcl
300
200
β tub56D
500
400
300

Figure S12b

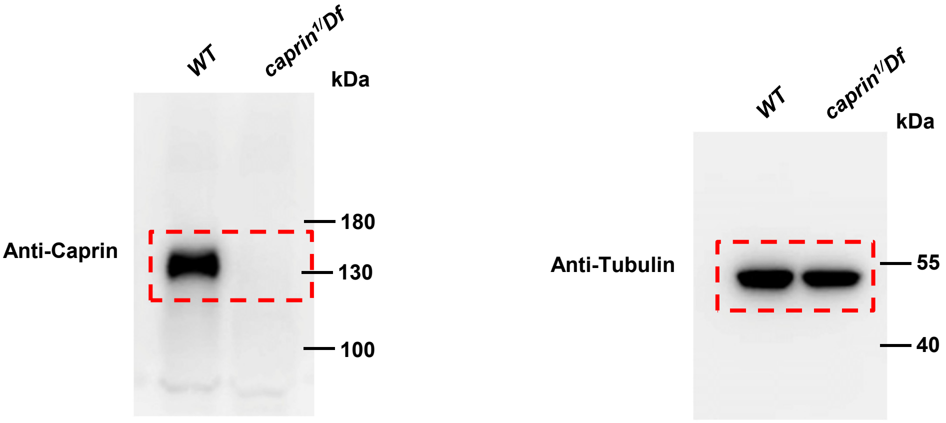

WT
caprin1/Df
kDa
Anti-Caprin
180
130
100
WT
caprin1/Df
kDa
Anti-Tubulin
55
40
